# A Deep Metric Learning and Multimodal Gated Fusion Framework for AI‐Driven Risk Assessment of Lingual Plate Perforation and Mandibular Canal Injury in Posterior Mandible Implants

**DOI:** 10.1155/ijod/5599213

**Published:** 2026-02-23

**Authors:** Khulood Ali Al-Taezi, Lin Liu, Abdulrahman Al-Badwi, Mohammed Ali Al-taezi, Shuo Dong, Mohammed Al-Habib, Chunbo Tang

**Affiliations:** ^1^ Department of Oral Implantology, The Affiliated Stomatological Hospital of Nanjing Medical University, Nanjing, 210000, China, njmu.edu.cn; ^2^ Jiangsu Key Laboratory of Oral Diseases, Nanjing Medical University, Nanjing, 210000, Jiangsu, China, njmu.edu.cn; ^3^ Jiangsu Province Engineering Research Centre of Stomatological Translational Medicine, Nanjing Medical University, Nanjing, Jiangsu, China, njmu.edu.cn; ^4^ State Key Laboratory Cultivation Base of Research, Prevention and Treatment for Oral Diseases, Nanjing Medical University, Nanjing, Jiangsu, China, njmu.edu.cn; ^5^ Faculty of Computers and Information Technology, Sana’a University, Sana’a, Yemen, su.edu.ye; ^6^ College of Intelligence and Computing, Tianjin University, Tianjin, 300350, China, tju.edu.cn; ^7^ School of Computer Science and Engineering, Central South University, Changsha, 410083, Hunan, China, csu.edu.cn

**Keywords:** AI, CBCT, deep learning, lingual plate perforation, mandibular canal injury, multimodal fusion, risk classification

## Abstract

**Objective:**

To provide an automated risk assessment for lingual plate perforation (LPP) and mandibular canal injury (MCI) in dental implants. Also, to reduce interoperator variability in risk classification and eliminate manual measurements during implant planning.

**Methods:**

A dataset of 896 CBCT‐implant records was used for training our model (DentaRisk‐Net). CBCT images were processed using ResNet‐18 encoders for each implant site view. Patient‐specific metadata was processed through a fully connected network. These modalities were fused using a gated fusion mechanism (GFM). Deep metric learning (DML) was employed to enhance distinguish of risk classes. The model was trained on two risk tasks: bone plate (BP) and mandibular canal (MC) tasks. Classification and regression metrics (accuracy, precision, recall, *F*1‐score, *R* ^2^, and ablation studies) were used to evaluate performance. True assessment rate (TAR) was used to assess the model’s agreement with human annotators. Grad‐CAM visualizations were conducted for qualitative evaluation.

**Results:**

The model achieved an accuracy exceeding 0.87 in detecting risk in implant sites, with precision and recall above 0.84. The GFM increased *F*1‐scores by 3%–5% and *R* ^2^ by 5%–8% for both risk tasks. The model achieved high performance for the Safe and Risk classes, whereas the Caution class was comparatively lower in BP and MC. TAR ≤1 values demonstrated acceptable alignment with human (BP: *r* = 0.63; MC: *r* = 0.72). Grad‐CAM visualizations confirmed the model’s ability for feature localization.

**Conclusion:**

By integrating imaging and metadata through DML and GFM, the model attempted to reduce interoperator variability in decision‐making and enhance risk‐classified assessment in the treatment planning process.

## 1. Introduction

In dental implants, the posterior mandible presents a particular risk due to the proximity of critical anatomical structures such as the mandibular canal (MC) and the lingual bone plate (BP) [1]. Improper evaluation or placement can result in lingual plate perforation (LPP) or MC injury (MCI), which may lead to irreversible consequences including paraesthesia, bleeding, or implant failure [1, 2]. Statistically, the incidence of LPP ranges between 1 and 2% or even higher [1, 3] and MCI up to 40% [1] (predominantly reflecting transient postoperative neurosensory disturbances rather than permanent injury) [4, 5]. Accurate potential risk identification is essential to ensure safe and successful implant placement [2, 6]. Traditionally, clinicians have relied on manual interpretation of CBCT images to assess anatomical suitability for implant procedures. This process involves evaluating multi‐view slices and manual labeling to assess anatomical features proximity, which is liable to interobserver variability [7]. Moreover, it requires substantial experience to correlate images with clinical decision‐making [8]. Recent developments in artificial intelligence (AI) and deep learning (DL) have opened new avenues for automating complex diagnostic tasks in dentistry and medicine. AI has demonstrated significant potential in improving medical discoveries by revealing hidden patterns and diagnostic cues within clinical data that often failed to be recognized by human clinicians [9]. Through multimodal and DL methodologies, AI systems are capable of analyzing complex imaging data, thereby enhancing diagnostic accuracy, optimizing treatment planning, and tailor‐made patient care [9]. Models using convolutional neural networks (CNNs) have demonstrated success in tasks such as lesion detection, root morphology analysis, and automated image landmarking [10–12]. CNN‐based classifiers are effective for image categorization tasks, but in the context of overlapping anatomical regions, the CNN can be found insufficient [13, 14]. Deep metric learning (DML) has been found effective to enhance class separability and has been proven effective for supervised classification tasks [15]. Likewise, multimodal gated fusion with dynamic weights between image features and structured metadata helps better approximating the clinician reasoning [9, 16]. However, the application of AI for anatomical risk assessment for dental implants, particularly in the posterior mandible, remains in its early stages [17, 18]. Moreover, most prior approaches were using either single‐view analysis or landmark labeling [19]. In this study, we introduce a novel framework that combines DML and the multimodal gated fusion mechanism (GFM) using CBCT images with structured patient‐specific metadata to assess the risk classes of surgical complications. This approach mimics the comprehensive reasoning process of clinicians who rely on both manual measurements and anatomical visualizations when planning implant procedures. Consequently, the model attempts to reduce variability in clinical decision‐making, streamline the treatment planning process, and classify enhance patient safety.

## 2. Materials and Methods

### 2.1. Patient Selection and Imaging Protocol

This retrospective study utilized a curated dataset comprising metadata from patients who underwent implant placement in posterior mandible sites. CBCT scans of patients obtained between 2020 and 2023 by a search of the electronic health records at the Jiangsu Hospital of Nanjing Medical University. The Affiliated Stomatological Hospital of Nanjing Medical University Ethical Committee Department approved retrieval and assessment of the CBCT scans for this study (Approval # PJ2020‐130‐001). The study was conducted with the guidelines laid out in the Declaration of Helsinki. Any patient’s data that can potentially breach data protection rules and regulations was exempted. Dataset were included posterior mandible implant planning records with complete CBCT scans. Any records with pathological mandibular alterations (e.g., cysts and tumors) or poor image quality due to artifacts or motion were excluded.

#### 2.1.1. CBCT Acquisition

CBCT scans were acquired using (Newtom VGi evo, Verona, Italy) with a standardized protocol: imaging parameters were set at 110 kVp, 5 mA, scan time 20 s and resolution 0.3 mm. Patients’ head positions had unified according to the horizontal and vertical reference lines created by the manufacturer before the images were taken. CBCT images of interest were reconstructed and loaded into Anatomage Invivo 7.0 virtual implant planning software (Anatomage, San Jose, CA, USA). All images were viewed in a dimly lit area on a 19‐inch flat panel screen with a 1920 × 1080 pixel resolution (HP Development Company, Palo Alto, Calif).

### 2.2. Data Preprocessing and Annotation

The dataset included 896 implant records extracted from DICOM files and virtual implant planning case reports. CBCT images were collected with dentate and missing mandibular first or second molars. CBCT scans were preoperative and virtual implant‐planning images. Virtual‐implant views were software‐generated planning views for implant placement. Therefore, the model predicts preoperative anatomical risk independent of operator decision. The average age of patients was 47.68 ± 14.97 years, with a range from 20 to 79 years. In each implant record, surgical outcomes and implant details were identified. The implant site was viewed in coronal, sagittal, and axial views before and after implant planning. Data was annotated by extracting the following:

#### 2.2.1. Structured Clinical Metadata

1. Tooth ID: anatomical location of the planned implant (e.g., #36–#47).
2. Implant size: diameter and length (Table S1).
3. Implant brand: manufacturer/series of implant used (Straumann Standard RN, WN; Active, Nobel Parallel CC) (Table S1).
4. Spatial coordinates (*x, y, z*) for critical landmarks:
  • Critical BP points (axial, sagittal, and coronal positions)
  • MC position (axial, sagittal, and coronal positions)

Spatial coordinates (*x, y, z*) were incorporated during model development to facilitate learning of implant position and anatomical relationships. These coordinates are not required during clinical inference, where risk prediction aimed to perform based on CBCT image inputs without manual landmarking.

5. Risk classes: to identify risk classes, anatomical feature measurements were defined as the following:
  • BP distance (BPD): linear distance from implant apex to closest BP.
  • MC distance (MCD): linear distance from implant apex to MC.

Based on BPD and MCD, risk classes were recorded as the following:

• Class 1 (safe): if MCD or BPD ≥3 mm
• Class 2 (caution): if 2 mm ≤ MCD or BPD <3 mm
• Class 3 (risk): if MCD or BPD <2 mm

MC distance and BP distance were used for ground‐truth risk labeling during dataset annotation. These measurements were not provided as input features to the model and were not available during inference. Risk classification was learnt from CBCT‐derived anatomical features, ensuring that no distance‐based values were directly accessible to the network.

Any missing or undefined fields were excluded from the final analysis. All identifiers were removed to maintain compliance with ethical research standards. Two implantologists independently annotated each case for LPP and MCI presence based on axial, coronal, and sagittal slices. Discrepancies were resolved through consensus. Images were cropped and resized using custom Python scripts for DICOM parsing, slice alignment, and resizing, ensuring standardized sample representation.

### 2.3. Model Architecture

**Figure 1:**
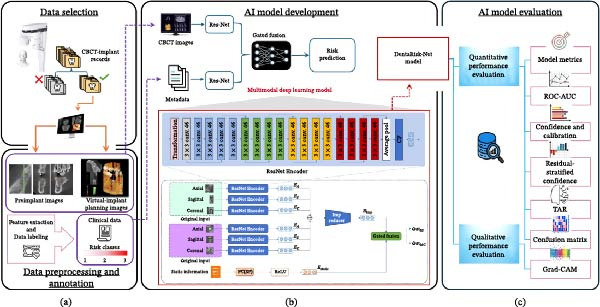
Schematic representation of the DentaRisk‐Net methodological workflow. The figure outlines the end‐to‐end process used in the development and evaluation of the DentaRisk‐Net model. It consists of: (a) Data Selection and Preprocessing, including pre and virtual‐implant images, features extraction, and annotation; (b) AI Model Development, where CBCT images and static data are processed through a multimodal gated fusion network incorporating ResNet encoders and a deep metric learning module for risk prediction; and (c) AI Model Evaluation, which assesses both quantitative metrics and qualitative outputs to validate the model’s performance and clinical interpretability.

The proposed model, DentaRisk‐Net, is a gated multimodal DL framework processed both radiographic images and patient‐specific metadata through specialized branches and fused both modalities’ representations using a gated mechanism. Six ResNet‐18 encoders were used (pretrained on ImageNet), each responsible for processing one of the six CBCT slices representing sagittal, coronal, and axial views in both preimplant and virtual‐implant views (Details in Supporting Information: Page 2 & 3). The key architectural components were depicted in Figure 1.

### 2.4. Training and Evaluation

The model was implemented in PyTorch and trained using an NVIDIA RTX 3090 GPU. An 80/10/10 split was applied to partition the data into training, validation, and test sets, classified by risk scores. Training was over 100 epochs with early stopping based on *F*1‐score on the validation set. Dropout layers with a rate of 0.3 were used after each encoder output to reduce overfitting. To evaluate the performance of the proposed DentaRisk‐Net model in risk assessment, experiments were conducted on two clinically critical tasks:

1. BP perforation risk (BP task).
2. MCI risk (MC task).

Multiple outcomes were conducted to evaluate model’s performance (Supporting Information: Page 4).

## 3. Results

### 3.1. Quantitative Performance Evaluation

#### 3.1.1. BP and MC Tasks Performance

Performance was benchmarked against ablated model variants (No‐DML, No‐Gate, Static‐Only, Image‐Only) using standard evaluation metrics. DentaRisk‐Net achieved superior performance on the BP classification task, yielding an *F*1‐score of 0.854, *R* ^2^ = 0.803, and MAE = 0.131, surpassing all ablated variants. Although No‐DML reached an *F*1 of 0.843, DentaRisk‐Net demonstrated better calibration and generalizability across all metrics (Table 1). On the MCI risk classification task, DentaRisk‐Net achieved an *F*1‐score of 0.830, *R* ^2^ = 0.835, and MAE = 0.128, outperforming No‐DML (*F*1 = 0.79), No‐Gate (*F*1 = 0.78), Static‐Only, and Image‐Only variants (Table 1).

**Table 1:** BP and MC ablated model variants metrics.

Model variant	Accuracy mean (SD)	Precision mean (SD)	Recall mean (SD)	*F*1‐score mean (SD)	*R* ^2^ mean (SD)	MAE mean (SD)	MSE mean (SD)
**BP**							
DentaRisk‐Net	0.89 (0.03)	0.86 (0.04)	0.84 (0.05)	0.85 (0.04)	0.80 (0.06)	0.13 (0.03)	0.02 (0.005)
No‐DML	0.88 (0.03)	0.84 (0.04)	0.84 (0.04)	0.84 (0.03)	0.77 (0.05)	0.14 (0.03)	0.03 (0.006)
No‐gate	0.86 (0.04)	0.83 (0.04)	0.81 (0.05)	0.82 (0.04)	0.75 (0.06)	0.14 (0.03)	0.02 (0.005)
Static only	0.85 (0.05)	0.81 (0.05)	0.80 (0.05)	0.80 (0.05)	0.74 (0.06)	0.15 (0.04)	0.03 (0.006)
Image only	0.84 (0.05)	0.82 (0.04)	0.79 (0.05)	0.80 (0.04)	0.73 (0.06)	0.16 (0.04)	0.03 (0.006)
**MC**							
DentaRisk‐Net	0.87 (0.03)	0.84 (0.03)	0.82 (0.04)	0.83 (0.03)	0.84 (0.05)	0.13 (0.03)	0.02 (0.004)
No‐DML	0.84 (0.04)	0.81 (0.04)	0.78 (0.05)	0.79 (0.04)	0.77 (0.06)	0.15 (0.03)	0.03 (0.006)
No‐Gate	0.83 (0.04)	0.80 (0.04)	0.78 (0.05)	0.78 (0.04)	0.76 (0.06)	0.15 (0.03)	0.03 (0.006)
Static only	0.82 (0.05)	0.78 (0.05)	0.74 (0.05)	0.76 (0.04)	0.74 (0.06)	0.15 (0.03)	0.03 (0.006)
Image only	0.83 (0.04)	0.80 (0.04)	0.77 (0.04)	0.78 (0.04)	0.74 (0.05)	0.15 (0.03)	0.03 (0.005)

#### 3.1.2. Class‐Wise Performance

Class‐wise performance metrics for BP and MC risk classification are summarized in Table 2. The model achieved high *F*1‐scores for the Safe and Risk classes, whereas performance for the Caution class was comparatively lower in both BP and MC risk assessments. Detailed tooth site‐specific performance results are presented in Table S2. The table presented key performance metrics for each tooth site (#36, #37, #46, and #47) in BPP and MCI prediction tasks. Regarding implant size, implant length showed clear changes across predicted risk classes. In the BP task, implant length mean increased from 10.2 ± 0.04 mm in Safe to 12.3 ± 0.04 mm in Risk cases. On the other hand, in the MC task, the increase was modest (10.8 ± 0.03 to 11.6 ± 0.06 mm). By contrast, implant diameter was relatively stable (≈4.5 mm) across risk classes in both tasks.

**Table 2:** BP and MC tasks class‐wise metrics.

Risk class	Task	Precision mean (SD)	Recall mean (SD)	*F*1‐score mean (SD)	Support
Safe	BP	0.91 (0.03)	0.89 (0.04)	0.90 (0.03)	90
	MC	0.95 (0.02)	0.90 (0.04)	0.93 (0.03)	84
Caution	BP	0.71 (0.07)	0.76 (0.06)	0.73 (0.06)	45
	MC	0.58 (0.08)	0.73 (0.07)	0.64 (0.07)	26
Risk	BP	0.93 (0.04)	0.91 (0.03)	0.92 (0.03)	44
	MC	0.94 (0.03)	0.90 (0.04)	0.92 (0.03)	69

#### 3.1.3. ROC‐AUC Analysis

The ROC‐AUC results for BP and MC risk classifications are illustrated in Figure 2a–d. Multiclass ROC analysis indicated strong class separability, with ROC‐AUC values for BP of 0.93 ± 0.03 for the Safe class, 0.86 ± 0.04 for the Caution class, and 0.90 ± 0.06 for the Risk class. Correspondingly, ROC‐AUC values for MC were 0.89 ± 0.05 (Safe), 0.77 ± 0.06 (Caution), and 0.90 ± 0.05 (Risk).

Figure 2ROC curves and regression plots for multi‐class model performance evaluation. ROC curves (left panels: a, b, c, and d illustrating the model’s diagnostic performance across different risk classes. Each ROC curve compares sensitivity (true positive rate) against 1‐specificity (false positive rate), with the red diagonal line representing AUC = 0.50. AUC provides a quantitative measure of the classifier’s discriminative ability, where values closer to 1 indicate superior performance. In the regression plots (right panels: e) data points are aligning at the top and closer to the diagonal line, indicating better calibration and prediction accuracy.

**Figure:**
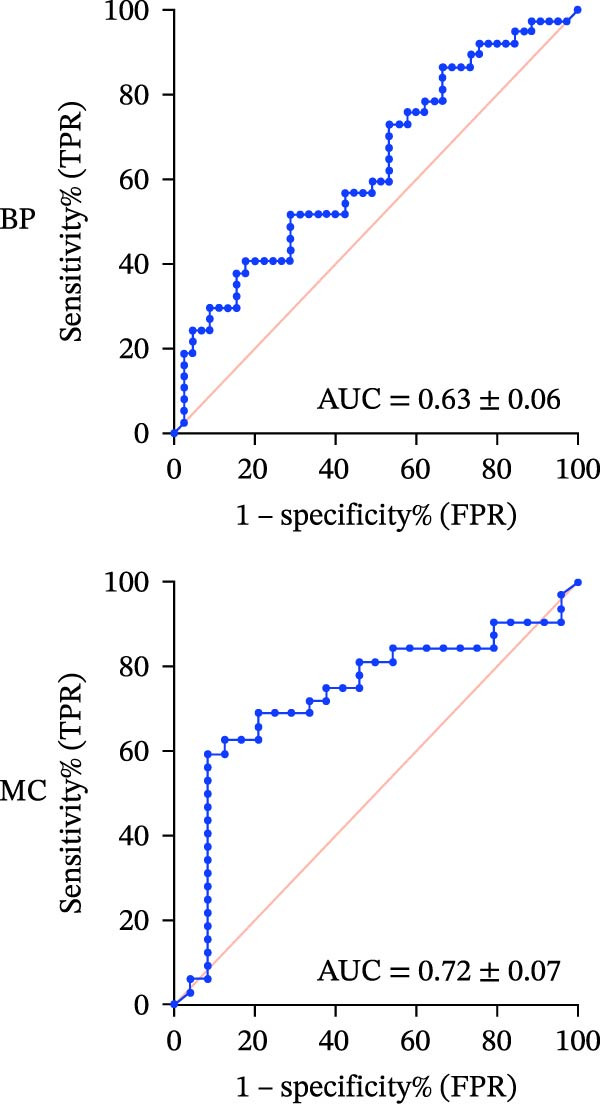
(a)

**Figure:**
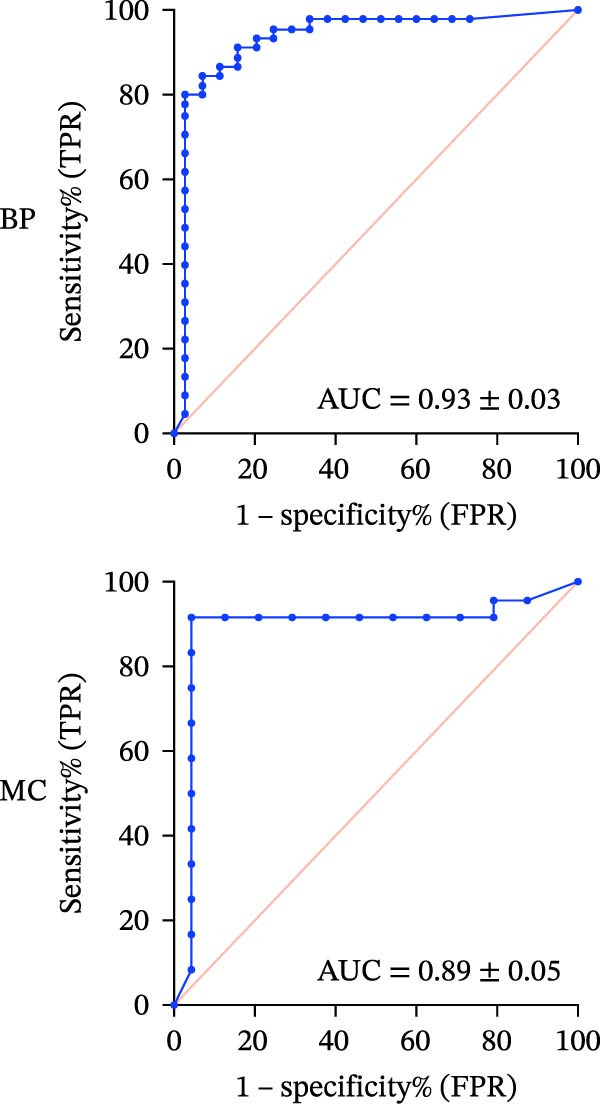
(b)

**Figure:**
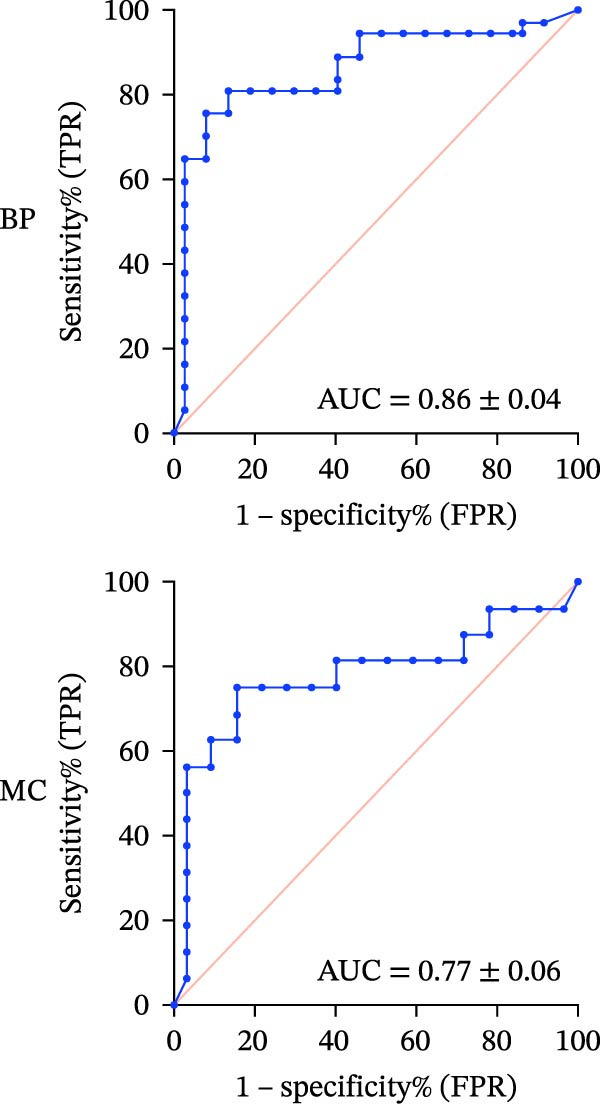
(c)

**Figure:**
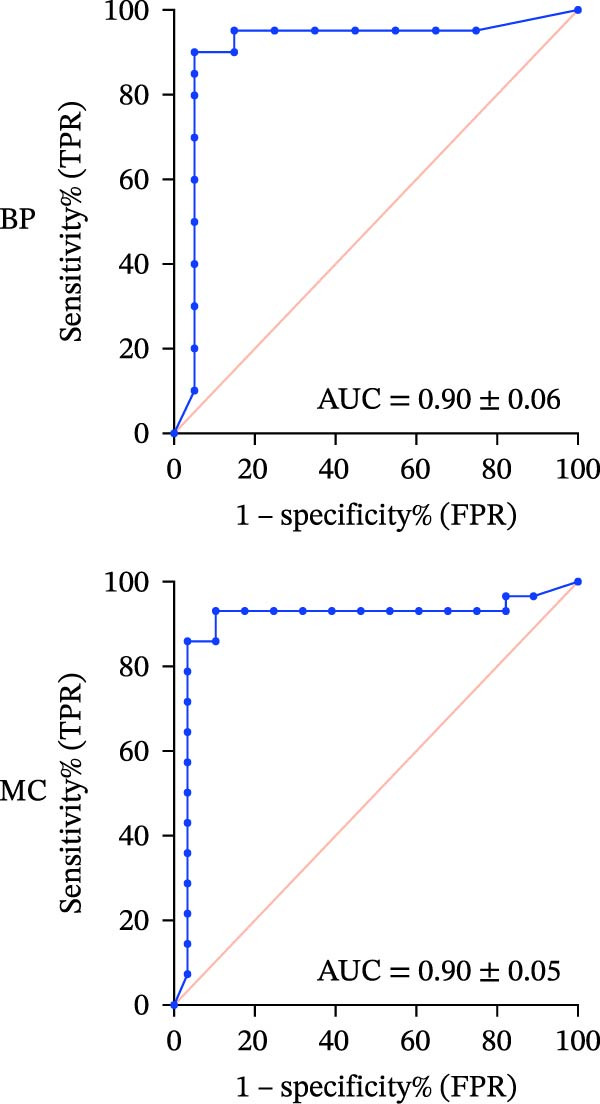
(d)

**Figure:**
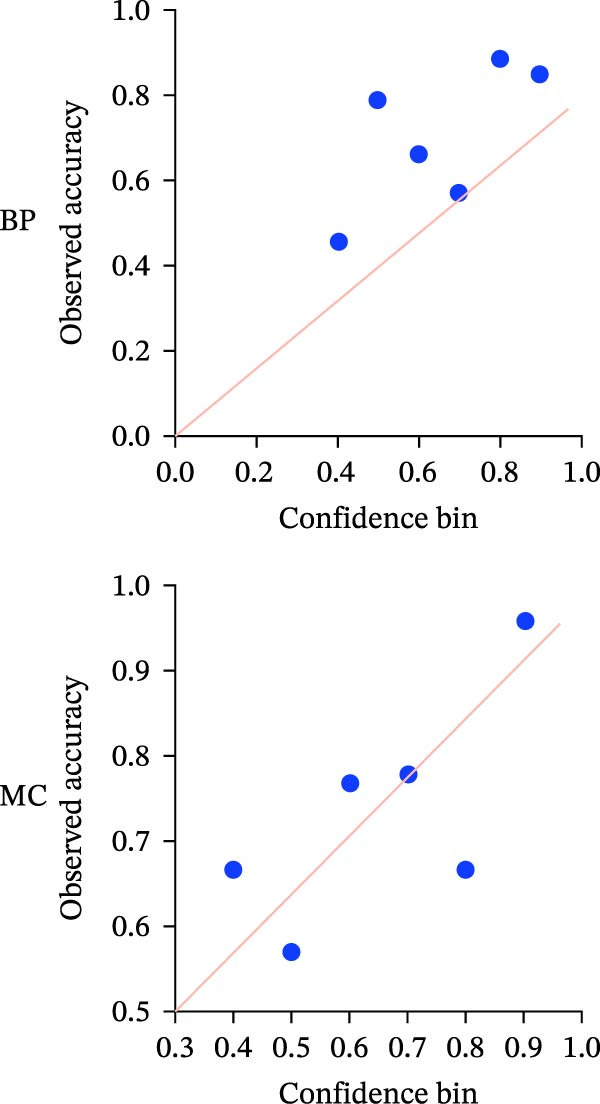
(e)

#### 3.1.4. Confidence, Calibration, and Residual‐ Stratified Confidence Visualizations

Calibration curves plotted predicted probabilities vs. observed accuracy across confidence bins, with an expected calibration error (ECE) of 0.038 for BP and 0.043 for MC (Figure 2e). These values indicate good calibration, as lower ECE means the predicted probabilities closely match the observed outcomes. In Figure 3 confidence histograms showed peaked distributions near 1.0 for correctly estimated Safe and Risk cases, with flatter distributions for Caution estimations, indicating higher uncertainty. Violin and box plots grouped by true and predicted labels for each class with overlaid box plots showing medians and interquartile ranges. Figure 3e, f shows predictions for Safe and Risk classes were tightly centered near high confidence values, while Caution exhibited wider variability than other classes. To further evaluate the relationship between model uncertainty and prediction error, we conducted a residual‐stratified confidence analysis. Residuals were defined as the difference between the model’s predicted continuous risk classes and the corresponding ground truth values. As illustrated in Figure 4, the plots present mean confidence values for the Safe, Caution, and Risk outputs across the (−1, 0.5) interval. Samples with small residuals (i.e., accurate predictions) exhibited higher average confidence scores, while those with larger residuals (indicating over or underprediction) showed reduced confidence (Figure 4).

Figure 3Model confidence distribution and class‐wise prediction analysis. Panel (a) shows the overall model confidence distribution for BP task predictions, while panel (b) displays the same for MC task predictions. Panels (c) and (d) illustrate the class‐wise confidence scores for BP and MC, respectively. In panels (c) and (d), blue represents the Safe class, red denotes the Caution class, and green indicates the Risk class. The plots visualize how prediction confidence is distributed across different risk classes, highlighting differences in uncertainty and prediction reliability for each risk class. Violin plots depict the density and distribution of model confidence scores for each risk class, (e) for BP and (f) for MC, grouped by true and predicted labels. This analysis reveals the model’s confidence behavior in correctly and incorrectly classified samples across different risk classes.

**Figure:**
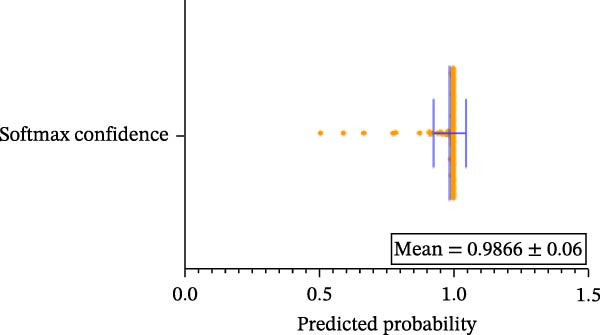
(a)

**Figure:**
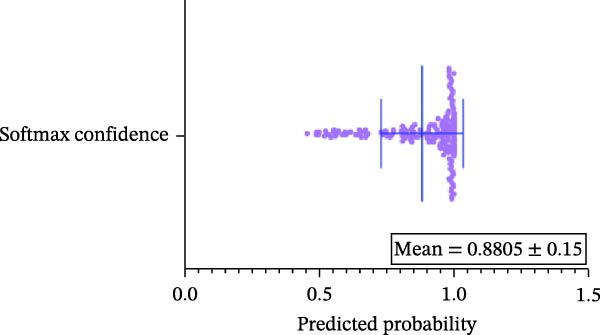
(b)

**Figure:**
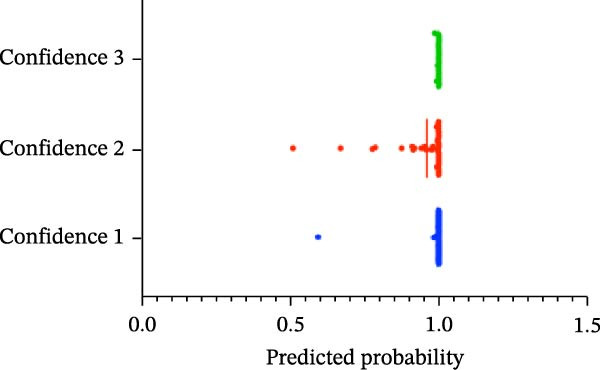
(c)

**Figure:**
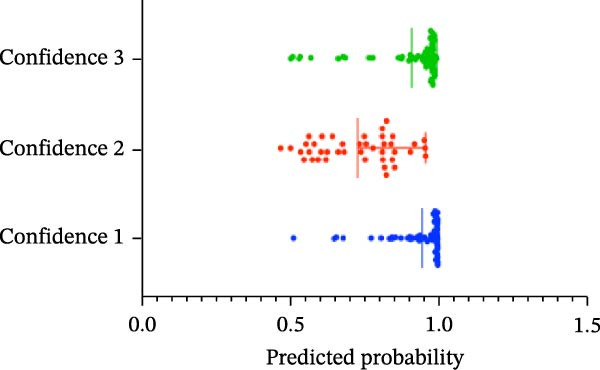
(d)

**Figure:**
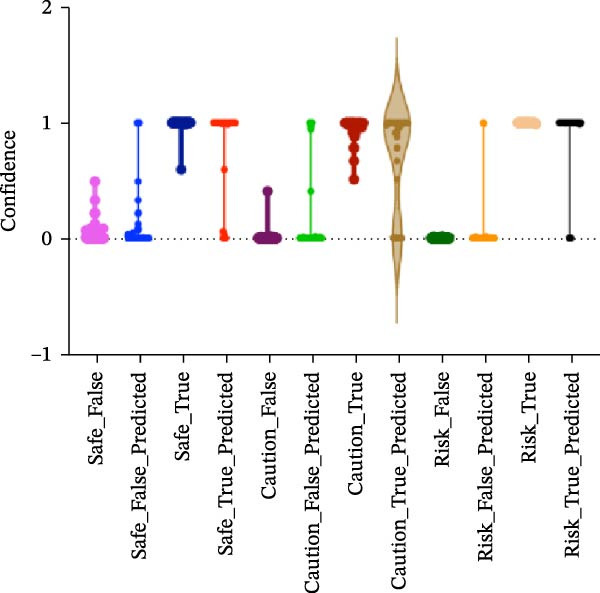
(e)

**Figure:**
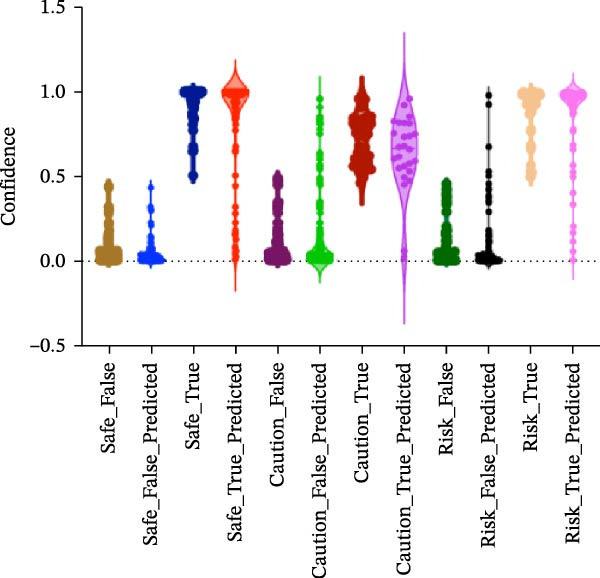
(f)

Figure 4Residual‐stratified confidence distribution for BP and MC predictions. Panels (a–c) correspond to BP predictions, illustrating the confidence–residual relationship for the safe (blue), caution (red), and risk (green) classes, respectively. Panels (d–f) correspond to MC predictions, shown in the same class order. Each panel demonstrates how model confidence varies with increasing residuals, where correctly classified samples with smaller residuals generally exhibit higher confidence, while misclassified cases show lower confidence. This residual‐based stratification provides an additional layer of reliability assessment for model predictions.

**Figure:**
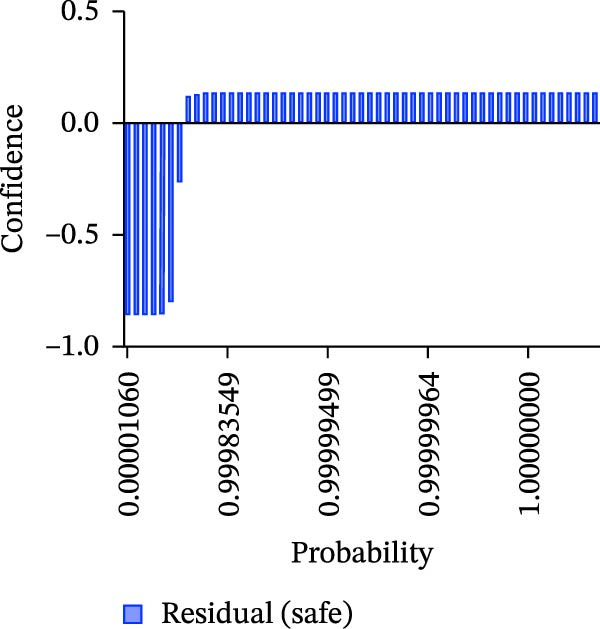
(a)

**Figure:**
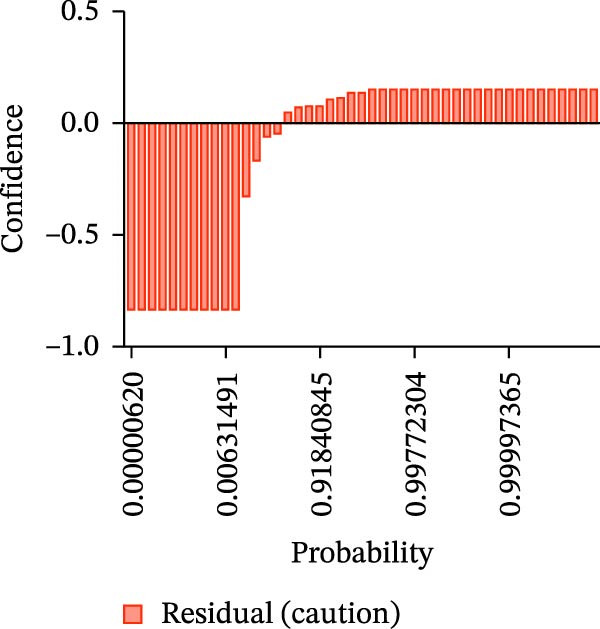
(b)

**Figure:**
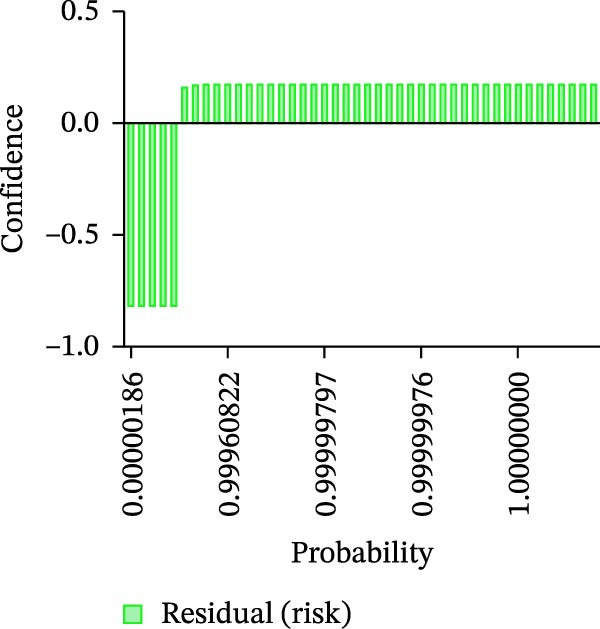
(c)

**Figure:**
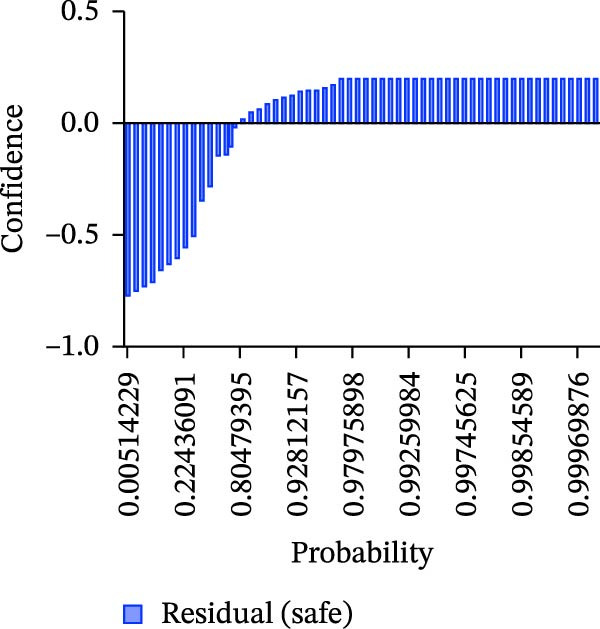
(d)

**Figure:**
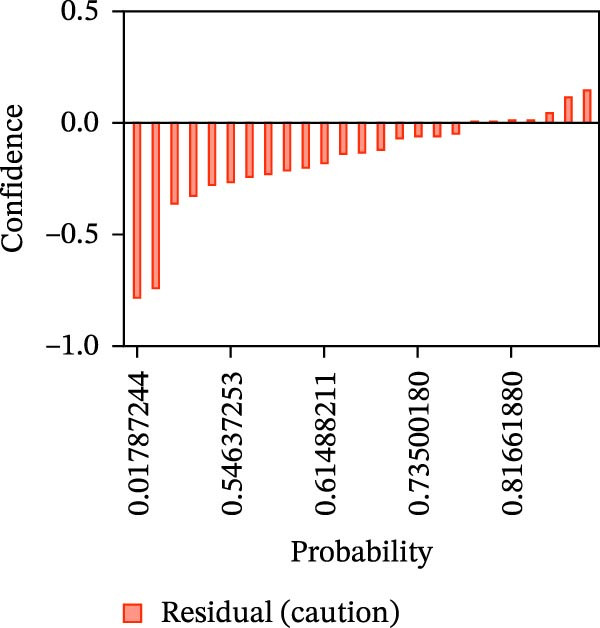
(e)

**Figure:**
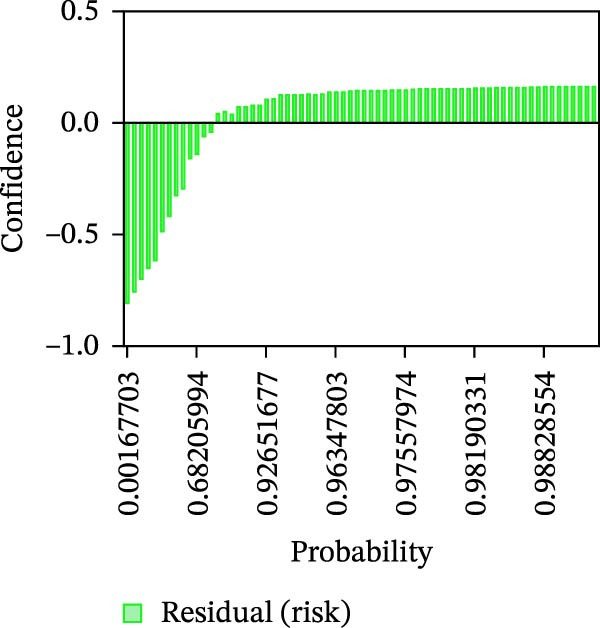
(f)

#### 3.1.5. Confusion Matrix Analysis

To evaluate the classification accuracy and misclassification patterns of the model, confusion matrices were generated for both the BP and MC (Figure 5). Each matrix illustrates how estimated class labels align with true class labels across the three risk levels: Safe (Class 1), Caution (Class 2), and Risk (Class 3). In the BP confusion matrix (Figure 5a), the model achieved high accuracy in predicting Safe (70/84) and Risk (62/69) classes, with most errors concentrated in the Caution group. Specifically, 13 Safe cases were misclassified as Caution, and seven Risk cases were also predicted as Caution. Conversely, the MC confusion matrix (Figure 5b) revealed stronger overall separability between classes. The model correctly predicted 80 out of 90 Safe cases, 34 out of 45 Caution cases, and 40 out of 44 Risk cases. Misclassifications were generally localized and symmetrical around the Caution class. Eight Caution cases were confused as Safe, and four Risk cases as Caution.

Figure 5Confusion matrices for BP and MC models in multi‐class implant risk classification. (a) Confusion matrix for the BP model, showing the distribution of predictions across three risk classes. The BP model correctly classified most samples in Class 1 (70) and Class 3 (62), with minor misclassifications primarily between Classes 1 and 2. (b) Confusion matrix for the MC model, demonstrating its classification performance across the same risk classes. The MC model achieved higher true positive counts in Class 1 (80) and Class 2 (34), with fewer misclassifications overall compared to the BP model.

**Figure:**
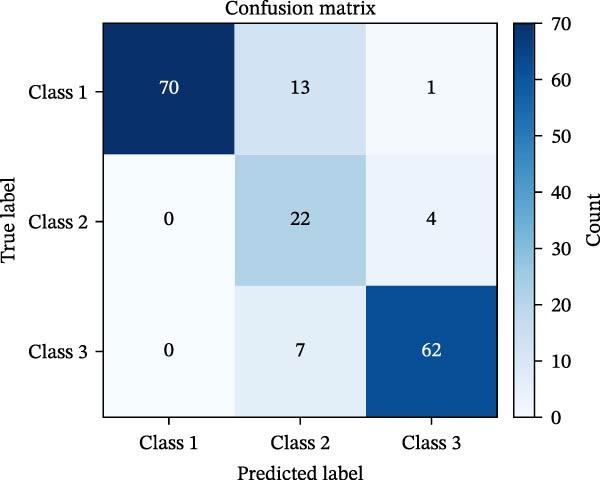
(a)

**Figure:**
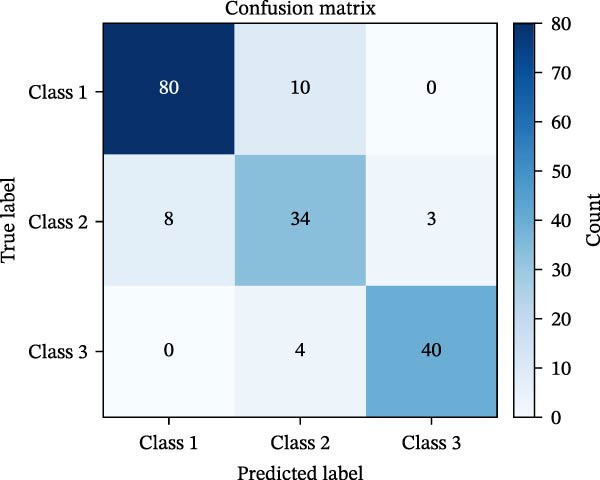
(b)

#### 3.1.6. True Assessment Rate (TAR)

BP task showed greater variability in high‐risk cases. As illustrated in the BP correlation matrix (Figure 6a), strong intraclass TAR correlation was observed between Safe and Caution (*r* = 0.63), whereas correlation between Safe and Risk was negligible (*r* = 0.41). This suggests that when the AI and human annotators disagreed, it was often due to misalignment in high‐risk classifications. This is further supported by the violin plots (Figure 6a), where the TAR ≤0 distribution for Risk is wide and flat, reflecting variability in exact‐match agreement, while TAR ≤1 remains relatively stable, indicating agreement within a one‐class margin is common and clinically acceptable. In contrast, MC task showed higher model‐human concordance. The MC correlation matrix (Figure 6b) demonstrates uniformly higher Pearson coefficients across class pairs: Safe‐Caution: *r* = 0.72, Safe‐Risk: *r* = 0.59, and Caution‐Risk: *r* = 0.43)

Figure 6Violin plots and correlation matrices of true assessment rate (TAR) for BP and MC models. (a) Distribution and Pearson correlation matrix of TAR thresholds (≤0, ≤1, and ≤2) for the BP model. The violin plots depict TAR variability across classes, while the correlation matrix shows moderate inter‐threshold associations, with the highest correlation between TAR ≤1 and TAR ≤2 (*r* = 0.57). (b) TAR distribution and correlation matrix for the MC model. ( ^∗^) *p*‐values indicate significant differences between TARs in human and AI assessment (Table S4).

**Figure:**
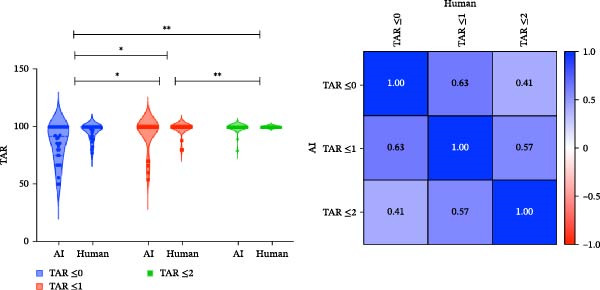
(a)

**Figure:**
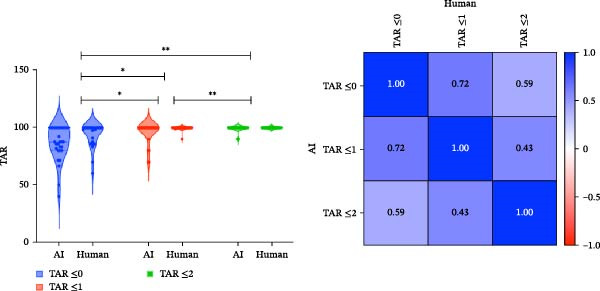
(b)

### 3.2. Qualitative Performance Evaluation

#### 3.2.1. Grad‐CAM Visualizations

The resulting attention maps were superimposed on CBCT slices. As illustrated in Figure 7, red zones denote regions of the model’s attention, indicating strong influence on the model’s decision, while blue areas correspond to lower relevance. The model consistently concentrated its attention on key anatomical structures, including related cortical bone and MC. This spatial focus aligns with known surgical risk zones for bone perforation or alveolar nerve injury. Across the “Precise Prediction” section (Table S3), the DentaRisk‐Net model classified risk for both BP and MC in alignment with the expert ground truth, with corresponding class probabilities showing high model confidence (e.g., >0.99 for correct predictions). Heatmaps consistently showed high activation near relevant anatomical landmarks: the lingual cortical margin for BP predictions and the inferior border for MC predictions.

Figure 7Grad‐CAM visualizations of model assessment accuracy for mandibular implant risk classification. Representative Grad‐CAM overlays from the DentaRisk‐Net model illustrating visual attention patterns across three categories: (a) Preise Prediction and (b) Misclassification. In precisely predicted cases, the model focused on clinically relevant anatomical structures, such as cortical bone and mandibular canal boundaries. In misclassified cases, Grad‐CAM maps showed lower readability for diagnostic landmarks. These visualizations support model transparency and help identify failure modes for clinical validation.

**Figure:**
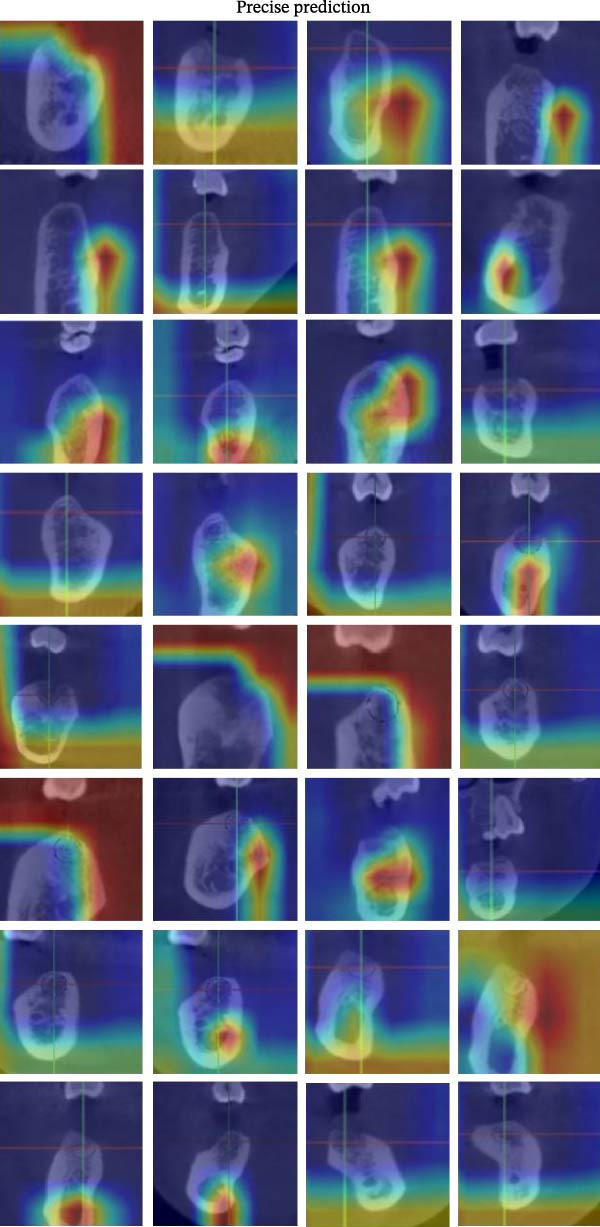
(a)

**Figure:**
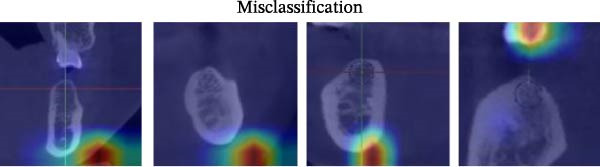
(b)

## 4. Discussion

Accurate assessment of anatomical risks such as lingual BP perforation and MCI is essential in posterior mandible implant planning to avoid complications like neurosensory damage, bleeding, and implant failure [1, 2, 6, 20]. Conventional assessment methods are susceptible to interobserver variability, as they rely on clinicians’ decisions [7]. In this study, we proposed DentaRisk‐Net, an AI framework that combines DML with a multimodal GFM for risk assessment from CBCT images without requiring segmentation and manual measurements. The model achieved an accuracy of 0.89 for BP and 0.87 for MC, with *F*1‐scores of 0.85 and 0.83, and Recall values of 0.84 and 0.82, respectively, outperforming several prior AI systems [21] (Table S4). For instance, Jeon et al. [22] reported only 78.65% accuracy for MC localization using a conventional CNN. While Al‐Sarem et al. [23] achieved high accuracy (90.81%) for tooth region detection, but their work was limited to classification and relied on ensembled model architectures. The comparable results in our model can be attributed to the synergistic contribution of DML and GFM modules. The GFM yielded consistent gains, increasing Accuracy, *F*1‐scores by 3%–5%, and *R* ^2^ by 5%–8% for both BP and MC risk classifications compared with models lacking fusion (Table 2). Compared to Elgarba et al. [18] who developed then validated [24] a cloud‐based implant planning system with 89%–95% accuracy, our fully automated model enhances workflow efficiency and eliminates error from segmentation bottlenecks. Similarly, the multistage system by Al‐Asali et al. [25] achieved high segmentation accuracy (Dice = 0.93), but required segmented ROI inputs, a limitation our model overcomes. Gong et al. [14] introduced a three‐stage framework for predicting inferior alveolar nerve (IAN) injury risk. Their model achieved high segmentation performance, Dice = 0.947 for mandibular third molar detection and accuracy = 0.846 for injury likelihood classification. Although clinically impactful, Gong et al.’s [14] approach is focused on nerve injury assessment and is related to 3rd molar classification tasks. Incorporating metadata into the model via MGF substantially improved generalization across anatomical and implant types, particularly in underrepresented variants. Models trained without fusion underperformed, especially during cross‐validation [16]. These findings reinforce earlier work on the value of integrating contextual metadata into radiographic tasks [26]. Using different implant sizes to predict risk class showed the influence of implant length in the model prediction, especially for bone perforation. Implant length clearly reduced BPD, thereby, elevated risk probability, while implant diameter alone provided less discriminative information. This pattern was more obvious in the BP model compared to MC, suggesting the model’s ability to recognize the distance to BP more than canal proximity. Despite DentaRisk‐Net comparable performance, the Caution risk class presented classification challenges, with greater variability compared to the Safe and Risk groups. This reflects findings from Lian et al. [13], who also reported decreased performance in intermediate caries categories due to overlapping features and resolution limitations. Similarly, imbalanced datasets have been shown to degrade classifier sensitivity and specificity, especially in multiclass scenarios. According to Aguilar‐Ruiz et al. [27] and Brzezinski et al. [28], who noted that ROC‐AUC may conceal subpar performance in minority classes, whereas precision–recall curves provide more detailed information on classifiers under imbalance. Further, ECE was consistently below 0.1 across tasks, confirming strong alignment between predicted probabilities and true outcomes. This is particularly relevant when compared to models like Alotaibi et al. [21], whose DL system struggled with accurate buccolingual placement and implant angulation, reporting up to 15.9 mm error margins and classification performance as low as 59% for diameter prediction (Table S4). These comparisons emphasize the strength of our DML‐enhanced embedding space for clinically grounded, discriminative representations.

Ensuring model transparency is critical for clinical deployment. We implemented Grad‐CAM to visualize attention maps during successful predictions. In accurate classifications (Figure 7, Table S3), activations were localized over cortical bone and the MC, consistent with clinical decision points. This aligns with the findings of prior literature [29–32], which demonstrated meaningful anatomical activation in root inclination, sinus abnormality prediction, radicular cysts and periapical granulomas detection, and predicting osteoporosis on mandibular cortical index tasks. In precise prediction cases (Table S3), the model exhibited high confidence, with dominant class probabilities frequently exceeding 0.98 and minimal competing class probabilities. In the “Misprediction” section, AI errors were primarily observed in borderline caution/risk zones, where BPD or MCD fell within 2–3 mm. Conversely, mispredicted cases demonstrated lower maximum probabilities (often <0.80) and greater probability dispersion across classes, reflecting model uncertainty in borderline anatomical scenarios. Such patterns underscore the potential value of integrating confidence‐based thresholds to identify cases for human review [33], thereby improving clinical reliability. However, mispredictions were often associated with implant size variation or anatomical overlap. Small changes in implant diameter occasionally shifted the predicted class boundary, indicating model sensitivity to borderline risk cases. This suggests that the model may benefit from multi‐resolution spatial encoding to better capture fine‐grained anatomical details. Expanding the dataset with balanced samples would minimize misprediction and enhance the model performance. To assess the consistency between prediction error and model confidence, we stratified residuals into discrete bins and analyzed the average predicted confidence for each class across residual intervals (<−0.5, −0.5–0, 0–0.5, and >0.5). High confidence and low residual agreement in the majority of Safe and Risk predictions increase the interpretability and dependability of the system, particularly in high‐stakes scenarios. Despite the strong overall performance of DentaRisk‐Net, classification of the intermediate “Caution” risk category remained more challenging compared to the Safe and Risk classes. This finding is not unexpected and reflects both dataset‐level and clinical factors. First, the Caution class was underrepresented (BP: *N* = 45; MC: *N* = 26), which limits the model’s ability to learn robust decision boundaries for intermediate risk scenarios. Second, the Caution category inherently represents a gray zone in implant planning, where anatomical distances fall near clinical thresholds (2–3 mm), resulting in overlapping visual features between adjacent risk classes. Such borderline cases are also known to exhibit higher interobserver variability among clinicians. Confidence and calibration analyses further revealed that the model expressed lower certainty and greater probability dispersion for Caution predictions, indicating appropriate uncertainty awareness rather than overconfident errors. This behavior suggests that Caution‐level predictions may be best interpreted as decision‐support outputs rather than definitive classifications. These findings align with earlier works [14, 34], which advocate for human‐AI collaboration in diagnostic ambiguity zones. DentaRisk‐Net showed >95% agreement with human annotations within ±1 risk class, particularly for MCI assessment. In contrast, higher disagreement in BP predictions may reflect interobserver variability in defining the cortical perforation threshold which is consistent with previous literature [23, 25, 31, 35]. The correlation analysis between TARs for Safe, Caution, and Risk classes reveals an AI prediction agreement with human expert assessments. The strong correlation between Safe and Caution categories in BP and MC tasks indicates comparable performance to clinicians in distinguishing lower‐risk scenarios. However, the weaker correlation between Safe and Risk classes, especially in BP assessment (*r* = 0.41), highlights areas where the AI model diverges from human judgments. This discrepancy suggests complex anatomical or clinical nuances in high‐risk classifications that challenge the model’s capture. Moderate‐to‐low correlations between Caution and Risk categories emphasize diagnostic difficulty in borderline high‐risk cases. These findings align with previous studies reporting AI struggles with intermediate‐to‐severe classifications due to limited training samples and overlapping imaging features [31, 35] This suggests that while the model performs reliably at lower‐risk thresholds, it may require further training or hybrid decision mechanisms to fully capture high‐risk anatomical complexity.

In this study, we leveraged CBCT images enriched with metadata to develop an AI model for risk‐classified assessment without requiring manual landmarks labeling which is prone to variability and often time‐consuming. This AI system demonstrated the ability to detect potential risk in implant sites, achieving an accuracy exceeding 87% across tested cases. Such an automated approach holds promise not only for improving risk prediction and surgical safety but also for transforming implant planning workflows. Improving this system could help in streamlining preoperative planning, reducing cognitive load, and enhancing patient outcomes in dental implants. Although the proposed model demonstrates promising performance, it was trained and evaluated using CBCT data from a single academic center and a single scanner platform. As mandibular anatomy and imaging characteristics may vary across populations and devices, multicenter external validation using heterogeneous datasets is necessary to establish broader generalizability and clinical robustness. Furthermore, the observed underperformance in intermediate risk class (Caution) is acknowledged as a limitation of the current study. It underscores the need for targeted class rebalancing, augmentation of borderline cases, and uncertainty‐guided human‐AI collaboration strategies to improve clinical utility in this critical decision zone. Additionally, the inclusion of multimodal inputs such as intraoral scans, clinical notes, and occlusion data could enrich contextual understanding and extend the model’s utility in dental implants.

## 5. Conclusion

DentaRisk‐Net offers an automated AI framework for risk‐classified assessment and implant site prediction in posterior mandible implants. Through integration of DML, gated fusion, CBCT imaging, and patient‐specific metadata; the model achieved acceptable accuracy, class‐wise reliability, and clinical relevance compared to prior approaches. With continued validation and refinement, it holds strong potential as a clinical decision‐support tool in dental implant surgery.

## Author Contributions

Study conceptualization: Khulood Ali Al‐Taezi and Chunbo Tang. Data curation and annotation: Khulood Ali Al‐Taezi, Lin Liu, and Shuo Dong. Methodology and software development: Abdulrahman Al‐Badwi, Mohammed Ali Al‐taezi, and Khulood Ali Al‐Taezi. Data analysis and validation: Khulood Ali Al‐Taezi, Lin Liu, Abdulrahman Al‐Badwi, and Mohammed Al‐Habib. Manuscript drafting: Khulood Ali Al‐Taezi, Abdulrahman Al‐Badwi, and Shuo Dong.

## Funding

This work was supported by the Jiangsu stomatological affiliated hospital of Nanjing Medical University.

## Disclosure

All authors revised the manuscript for important intellectual content and approved the final version of the article. Any AI‐assisted language edits were thoroughly reviewed and approved by the authors.

## Conflicts of Interest

The authors declare no conflicts of interest.

## Supporting Information

Additional supporting information can be found online in the Supporting Information section.

## Supporting information


**Supporting Information** Methods: (Model Architecture): A detailed description of the DentaRisk‐Net framework is provided. The section elaborates on (1) the six ResNet‐18 image encoders for multi‐view CBCT feature extraction, (2) the static metadata encoder for integrating clinical and implant variables, (3) the gated fusion mechanism for adaptive multimodal feature weighting, (4) the output layer for risk score generation, and (5) the deep metric learning regularization strategy to enhance embedding separability. In addition, the Supplementary Methods include definitions of all evaluation metrics (Accuracy, Precision, Recall, F1‐score, MAE, MSE, *R*², ROC‐AUC, ECE, and TAR), ablation study design, and Grad‐CAM visualization procedures for interpretability assessment. Table S1: Implant size options categorized by manufacturer and series used for preoperative planning. Table S2: Tooth‐site‐wise performance metrics of DentaRisk‐Net. Table S3: Grad‐CAM visual case‐class analysis for representative implant sites. Table S4: Summary of studies on AI‐driven methods for dental implant planning, risk assessment, and anatomical structure detection. Table S5: Comparison of True Assessment Rates (TAR) between AI predictions and human expert assessments for LPP and MCI.

## Data Availability

The data that support the findings of this study are available from the corresponding author upon reasonable request.
