# Supplementary material for: A Deep Metric Learning and Multimodal Gated Fusion Framework for AI‐Driven Risk Assessment of Lingual Plate Perforation and Mandibular Canal Injury in Posterior Mandible Implants
Source: Int J Dent. 2026 Feb 23;2026:5599213. doi: 10.1155/ijod/5599213 (PMC12927901; doi:10.1155/ijod/5599213)
Supplement: Supplementary file 1 — Supporting Information Methods: (Model Architecture): A detailed description of the DentaRisk‐Net framework is provided. The section elaborates on (1) the six ResNet‐18 image encoders for multi‐view CBCT feature extraction, (2) the static metadata encoder for integrating clinical and implant variables, (3) the gated fusion mechanism for adaptive multimodal feature weighting, (4) the output layer for risk score generation, and (5) the deep metric learning regularization strategy to enhance embedding separability. In addition, the Supplementary Methods include definitions of all evaluation metrics (Accuracy, Precision, Recall, F1‐score, MAE, MSE, R², ROC‐AUC, ECE, and TAR), ablation study design, and Grad‐CAM visualization procedures for interpretability assessment. Table S1: Implant size options categorized by manufacturer and series used for preoperative planning. Table S2: Tooth‐site‐wise performance metrics of DentaRisk‐Net. Table S3: Grad‐CAM visual case‐class analysis for representative implant sites. Table S4: Summary of studies on AI‐driven methods for dental implant planning, risk assessment, and anatomical structure detection. Table S5: Comparison of True Assessment Rates (TAR) between AI predictions and human expert assessments for LPP and MCI. [file IJOD-2026-5599213-s001.docx]

**Table S1.** Implant size options by manufacturers and series

| **Manufacturer** | **Series** | **Size** | | |
| --- | --- | --- | --- | --- |
|  |  | **Diameter (mm)** | **Length (mm)** |  |
| Straumann | Standard (RN) | 3.3 | 8-10-12-14 | |
|  |  | 4.1 | 8-10-12-14 | |
|  |  | 4.8 | 8-10-12-14 | |
|  | Standard (WN) | 4.8 | 8-10-12-14 | |
| Nobel Biocare | Active (RP) | 3.5 | 8.5-10-11.5-13 | |
|  |  | 4.3 | 8.5-10-11.5-13 | |
|  |  | 5 | 8.5-10-11.5-13 | |
|  |  | 5.5 | 8.5-10-11.5-13 | |
|  | Parallel CC (RP) | 3.75 | 8.5-10-11.5-13 | |
|  |  | 4.3 | 8.5-10-11.5-13 | |
|  |  | 5 | 8.5-10-11.5-13 | |
|  |  | 5.5 | 8.5-10-11.5-13 | |

This table summarizes the commercially available dental implant sizes used in the study, categorized by manufacturer and series. Each row presents a combination of implant diameter (mm) and the corresponding available lengths (mm). These configurations represented the selection variability was used during preoperative planning for posterior mandibular implant placement. (RN); Regular Neck. (WN); Wide Neck. (RP); Regular Platform. CC; Conical Connection.

**Table S2. Tooth site-wise performance metrics of DentaRisk model.**

| **Tooth Site** | **Accuracy**  **Mean (SD)** | **Precision**  **Mean (SD)** | **Recall**  **Mean (SD)** | **F1-score**  **Mean (SD)** | **MAE**  **Mean (SD)** | **MSE**  **Mean (SD)** | **R²**  **Mean** |
| --- | --- | --- | --- | --- | --- | --- | --- |
| **BPP** |  |  |  |  |  |  |  |
| **#46** | 0.84 (0.36) | 0.84 (0.1) | 0.84 (0.1) | 0.84 (0.1) | 0.16 (0.36) | 0.16 (0.36) | 0.78 |
| **#47** | 0.8 (0.4) | 0.8 (0.07) | 0.8 (0.18) | 0.79 (0.08) | 0.2 (0.4) | 0.2 (0.4) | 0.55 |
| **#36** | 0.92 (0.26) | 0.93 (0.09) | 0.92 (0.03) | 0.93 (0.04) | 0.08 (0.26) | 0.08 (0.26) | 0.86 |
| **#37** | 0.76 (0.42) | 0.92 (0.31) | 0.76 (0.13) | 0.81 (0.16) | 0.24 (0.42) | 0.24 (0.42) | 0.73 |
| **MCI** |  |  |  |  |  |  |  |
| **#46** | 0.91 (0.29) | 0.94 (0.29) | 0.91 (0.29) | 0.92 (0.29) | 0.09 (0.29) | 0.09 (0.29) | 0.89 |
| **#47** | 0.75 (0.44) | 0.76 (0.44) | 0.75 (0.44) | 0.75 (0.44) | 0.30 (0.57) | 0.40 (0.94) | 0.37 |
| **#36** | 0.89 (0.32) | 0.91 (0.32) | 0.89 (0.32) | 0.90 (0.32) | 0.11 (0.32) | 0.11 (0.32) | 0.82 |
| **#37** | 0.65 (0.49) | 0.62 (0.49) | 0.65 (0.49) | 0.60 (0.49) | 0.35 (0.49) | 0.35 (0.49) | 0.56 |

**2.3 Model Architecture**

The proposed model, DentaRisk-Net, is a gated multimodal deep learning framework designed for automated prediction of LPP and MCI risks. It processed both radiographic images and patient-specific metadata through specialized branches and fused both modalities’ representations using a gated mechanism. The key architectural components were as follows:

**2.3.1 Image encoders**

Six ResNet-18 encoders were used (pretrained on ImageNet), each responsible for processing one of the six CBCT slices representing sagittal, coronal, and axial views, in both pre-implant and virtual-implant views. The final fully connected layer of each ResNet was removed, so each encoder outputs a deep feature map which was flattened and concatenated across all views, resulting in a combined high-dimensional imaging feature vector (Fig. 1). This process can be expressed in the following formula:


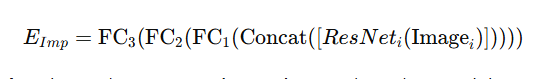


Where $E_{Imp}$ is the representation of the images modality, ${FC}_{i}$ is a fully connected network and $i$ is the order of output dimension $R= \{ 1024 , 512, 64 \}$ and Concan is the concatenation operation.

**2.3.2 Static metadata encoder**

Structured clinical and implant metadata (e.g., implant dimensions, anatomical measurements (BPD and MCD), spatial coordinates of bone and nerve landmarks) were passed through a fully connected feedforward network (FC) with two linear layers and ReLU activations function. This network compressed the 17-dimensional input into a 64-dimensional latent vector representing the static patient-specific context $E_{Static}$. This component is referred as the Image Implant Reducer in the architectural diagram (Fig. 1 main manscript).

**2.3.3 Gated fusion mechanism**

To dynamically weigh the contributions of imaging and static data, a gated fusion mechanism is employed. A sigmoid gate is computed from the reduced imaging vector:


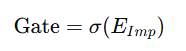


Where σ is the sigmoid function. This gate modulates the fusion of the two modalities as follows:


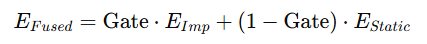


Where
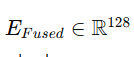
 is fused embedding and R has dimension of 128 ( 64 from image and 64 from metadata). This gating mechanism learns patient-specific fusion weights, dynamically balancing the contributions of image and metadata features according to their relevance in each clinical context.

**2.3.4 Output layer**

The fused embedding
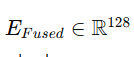
 (64 from image, 64 from metadata) was passed through a final linear layer to generate the output:


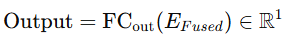


Where ${EFC}_{out}$ has a fully connected feedforward network followed by a sigmoid activation function. This scalar output represented the predicted risk score in terms of classification.

**2.3.5 Deep metric learning regularization**

To enhance feature discriminability, the model integrated Triplet Margin Loss using cosine similarity and semi-hard triplet mining during training. This loss encouraged the embedding space to group similar risk levels closer together while separating dissimilar ones, improving the model’s generalization to subtle anatomical variations.

**The following outcomes were conducted to evaluate the model’s performance:**

**Regression and classification models metrics:**

**Accuracy:** The number of correct predictions out of total number of predictions

**Precision:** The number of predicted values that are actually correct.

**Recall (Sensitivity):** The number of actual values correctly predicted

**F1-score:** The harmonic mean of precision and recall.

**Mean Absolute Error (MAE):** The average magnitude of the errors between predicted and actual values.

**Mean Squared Error (MSE):** The average of the squared differences between predicted and actual values. By squaring the errors, it penalizes larger errors more heavily than MAE.

**Coefficient of Determination (R²):** The proportion of variance in the observed data that is explained by the model’s predictions [1].

**Ablation studies:** To investigate the contribution of each architectural component by removing key components such as DML and the gated fusion mechanism, and by individual modalities (only images or only static data). Each ablation model was retrained under the same settings, and its performance was compared to the full model to quantify performance degradation.

**Receiver Operating Characteristic - Area Under the Curve (ROC-AUC):** The ROC graphical plot illustrates the diagnostic ability of a classifier by plotting the True Positive Rate (TPR) against the False Positive Rate (FPR) at various threshold settings [1].

TPR (Sensitivity) = TP / (TP + FN)

FPR = FP / (FP + TN)

AUC  under the ROC curve quantifies the overall ability of the model to discriminate between classes, independent of any specific decision threshold [1].

**Confusion Matrix:** measures how well a classification model performs by comparing the model’s outputs (i.e., predicted classes) against the actual ground truth labels [2].

**Model Confidence:** The probability that the model assigns to its predicted class. Confidence score value between 0 and 1 reflects how certain the model about its prediction [2].

**Calibrations:** evaluates how well the model’s predicted confidence aligns with actual accuracy. Expected Calibration Error (ECE) measures the average difference between predicted confidence and actual accuracy across bins of predictions [3].

**True Assessment Rate (TAR):** To evaluate the agreement between model predictions and expert clinical judgment, we employed a stratified TAR framework across three ordinal risk classes (Safe–Caution–Risk). TAR ≤ 0 denoted exact class matches, TAR ≤ 1 allowed for a one-class deviation (e.g., Risk predicted as Caution), and TAR ≤ 2 encompassed all predictions within the ordinal structure. Human reference labels were provided by two certified implantologists. Each annotator independently assessed the CBCT scans and corresponding implant metadata according to risk classes mentioned in Data Preprocessing and annotation. Discrepancies between annotators were resolved through consensus review in a joint session. Class-wise TAR scores were computed for both the AI model and human annotators. Pearson correlation coefficients were calculated to quantify the strength of agreement between AI and human assessments.

**Visual interpretability via Gradient-weighted Class Activation Mapping (Grad-CAM):** To qualitatively assess the anatomical regions contributing to the AI model’s assessment of risk. The Grad-CAM technique works by computing the gradient of the predicted class probability and applied to the final convolutional layer of the model image encode[4]. DentaRisk-Net’s gradients of the assigned SoftMax score for the target risk class was backpropagated with respect to the selected layer. Gradients were globally average-pooled to obtain weights, which are subsequently combined with the corresponding feature maps to generate a coarse localization map highlighting regions most influential in the model’s predictions. The resulting heatmap was up sampled and overlaid on the original CBCT slice, providing a visual explanation of model attention

**Table S3.** **Visual Case-Class Analysis (GRAD-CAM heatmaps).**

|  | **Precise Prediction** | | | | | | | | | | | | | | | | | | | | | | | | | | |
| --- | --- | --- | --- | --- | --- | --- | --- | --- | --- | --- | --- | --- | --- | --- | --- | --- | --- | --- | --- | --- | --- | --- | --- | --- | --- | --- | --- |
| **Tooth Site** | #46 | | | | | 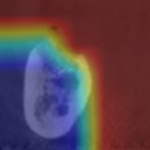 | #46 | | | | | | | 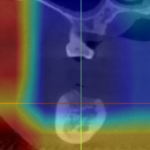 | #46 | | | | 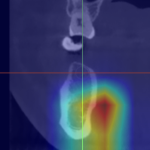 | #46 | | | | | | | 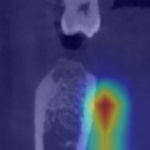 |
| **Implant Size** | 5*13 mm | | | | |  | 5*13 mm | | | | | | |  | 4.8*10 mm | | | |  | 4.1*10 mm | | | | | | |  |
| **Actual Risk** | Class (3): Risk | | | | |  | Class (2): Caution | | | | | | |  | Class (2): Caution | | | |  | Class (1): Safe | | | | | | |  |
| **Predicted Risk** | **BP** | | **MC** | | |  | **BP** | | | **MC** | | | |  | **BP** | | **MC** | |  | **BP** | | | | | **MC** | |  |
|  | 3 | | - | | |  | - | | | 2 | | | |  | 2 | | - | |  | 1 | | | | | - | |  |
| **Probabilities** | **Class (1)** | **Class (2)** | | | **Class (3)** |  | **Class (1)** | **Class (2)** | | | | **Class (3)** | |  | **Class (1)** | **Class (2)** | | **Class (3)** |  | **Class (1)** | | **Class (2)** | | | | **Class (3)** |  |
|  | 0.0087 | 0.0450 | | | 0.9461 |  | 9.920e-05 | 0.9999 | | | | 1.400e-05 | |  | 2.157e-3 | 0.9977 | | 1.194e-4 |  | 0.9985 | | 0.0014 | | | | 1.11e-06 |  |
| **BPD** | 0 mm | | | | |  | 0 mm | | | | | | |  | 2.7 mm | | | |  | 3.25 mm | | | | | | |  |
| **MCD** | 2 mm | | | | |  | 2 mm | | | | | | |  | 8.8 mm | | | |  | 6.29 mm | | | | | | |  |
| **Tooth Site** | #36 | | | | | 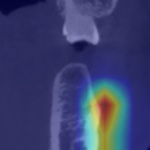 | #36 | | | | | | | 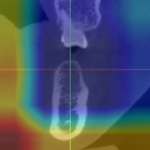 | #36 | | | | 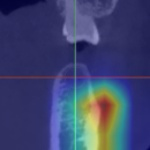 | #46 | | | | | | | 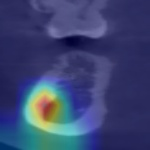 |
| **Implant Size** | 4.3*8.5 mm | | | | |  | 4.3*8.5 mm | | | | | | |  | 4.3*13 mm | | | |  | 5*11.5 mm | | | | | | |  |
| **Actual Risk** | Class (1): Safe | | | | |  | Class (1): Safe | | | | | | |  | Class (2): Caution | | | |  | Class (2): Caution | | | | | | |  |
| **Predicted Risk** | **BP** | | **MC** | | |  | **BP** | | | **MC** | | | |  | **BP** | | **MC** | |  | **BP** | | | | | **MC** | |  |
|  | 1 | | - | | |  | - | | | 1 | | | |  | 2 | | - | |  | - | | | | | 2 | |  |
| **Probabilities** | **Class (1)** | **Class (2)** | | | **Class (3)** |  | **Class (1)** | **Class (2)** | | | | **Class (3)** | |  | **Class (1)** | **Class (2)** | | **Class (3)** |  | **Class (1)** | | **Class (2)** | | | | **Class (3)** |  |
|  | 0.9975 | 0.0025 | | | 8.110e-7 |  | 0.9999 | 1.130e-6 | | | | 6.150e-11 | |  | 1.350e-5 | 0.99997 | | 1.280e-5 |  | 0.2224 | | 0.7769 | | | | 0.0006 |  |
| **BPD** | 3.31 mm | | | | |  | 3.31 mm | | | | | | |  | 2.45 mm | | | |  | 3.4 mm | | | | | | |  |
| **MCD** | 10 mm | | | | |  | 10 mm | | | | | | |  | 6.21 mm | | | |  | 2.16 mm | | | | | | |  |
| **Tooth Site** | #36 | | | | | 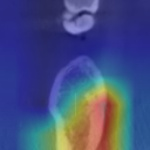 | #36 | | | | | | | 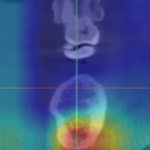 | #36 | | | | 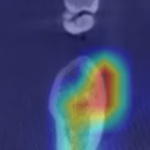 | #46 | | | | | | | 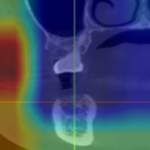 |
| **Implant Size** | 4.8*10 mm | | | | |  | 4.3*8.5 mm | | | | | | |  | 4.8*10 mm | | | |  | 4.3*10 mm | | | | | | |  |
| **Actual Risk** | Class (1): Safe | | | | |  | Class (1): Safe | | | | | | |  | Class (1): Safe | | | |  | Class (1): Safe | | | | | | |  |
| **Predicted Risk** | **BP** | | **MC** | | |  | **BP** | | | **MC** | | | |  | **BP** | | **MC** | |  | **BP** | | | | | **MC** | |  |
|  | - | | 1 | | |  | - | | | 1 | | | |  | 1 | | - | |  | 1 | | | | | - | |  |
| **Probabilities** | **Class (1)** | **Class (2)** | | | **Class (3)** |  | **Class (1)** | **Class (2)** | | | | **Class (3)** | |  | **Class (1)** | **Class (2)** | | **Class (3)** |  | **Class (1)** | | **Class (2)** | | | | **Class (3)** |  |
|  | 00.9905 | 0.0094 | | | 1.78e-06 |  | 0.9547 | 0.0452 | | | | 3.16e-05 | |  | 0.9999 | 5.71e-05 | | 3.24e-08 |  | 0.9999 | | 1.07e-05 | | | | 9.39e-11 |  |
| **BPD** | 3.79 mm | | | | |  | 4.39 mm | | | | | | |  | 3.79 mm | | | |  | 3.65 mm | | | | | | |  |
| **MCD** | 3.86 mm | | | | |  | 6.5 mm | | | | | | |  | 3.86 mm | | | |  | 1.29 mm | | | | | | |  |
| **Tooth Site** | #36 | | | | | 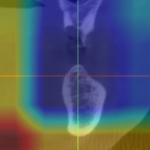 | #36 | | | | | | | 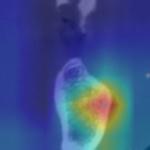 | #46 | | | | 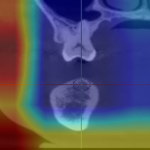 | #46 | | | | | | | 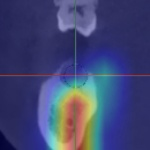 |
| **Implant Size** | 4.1*8 mm | | | | |  | 4.1*10 mm | | | | | | |  | 5*8.5 mm | | | |  | 4.8*14 mm | | | | | | |  |
| **Actual Risk** | Class (1): Safe | | | | |  | Class (1): Safe | | | | | | |  | Class (1): Safe | | | |  | Class (3): Risk | | | | | | |  |
| **Predicted Risk** | **BP** | | **MC** | | |  | **BP** | | | **MC** | | | |  | **BP** | | **MC** | |  | **BP** | | | | **MC** | | |  |
|  | - | | 1 | | |  | 1 | | | - | | | |  | 1 | |  | |  | - | | | | 3 | | |  |
| **Probabilities** | **Class (1)** | **Class (2)** | | | **Class (3)** |  | **Class (1)** | **Class (2)** | | | | **Class (3)** | |  | **Class (1)** | **Class (2)** | | **Class (3)** |  | **Class (1)** | **Class (2)** | | | | | **Class (3)** |  |
|  | 0.9307 | 0.06910 | | | 0.0001 |  | 0.9999 | 5.86e-09 | | | | 1.50e-13 | |  | 0.9999 | 4.96e-06 | | 8.48e-11 |  | 1.53e-09 | 9.20e-08 | | | | | 0.9999 |  |
| **BPD** | 3.31 mm | | | | |  | 3.51 mm | | | | | | |  | 3.03 mm | | | |  | 0 mm | | | | | | |  |
| **MCD** | 7.95 mm | | | | |  | 5.82 mm | | | | | | |  | 2.25 mm | | | |  | 1.68 mm | | | | | | |  |
| **Tooth Site** | #47 | | | | | 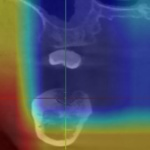 | #47 | | | | | | | 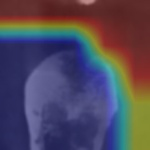 | #46 | | | | 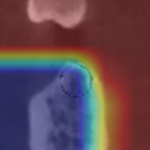 | #46 | | | | | | | 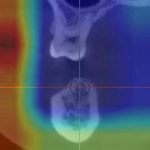 |
| **Implant Size** | 4.8*13 mm | | | | |  | 4.3*10 mm | | | | | | |  | 4.8*8 mm | | | |  | 4.8*8 mm | | | | | | |  |
| **Actual Risk** | Class (3): Risk | | | | |  | Class (1): Safe | | | | | | |  | Class (1): Safe | | | |  | Class (2): Caution | | | | | | |  |
| **Predicted Risk** | **BP** | | **MC** | | |  | **BP** | | | | **MC** | | |  | **BP** | | **MC** | |  | **BP** | | | | **MC** | | |  |
|  | **-** | | 3 | | |  | 1 | | | | - | | |  | 1 | | - | |  | - | | | | 2 | | |  |
| **Probabilities** | **Class (1)** | **Class (2)** | | | **Class (3)** |  | **Class (1)** | | **Class (2)** | | | | **Class (3)** |  | **Class (1)** | **Class (2)** | | **Class (3)** |  | **Class (1)** | | | **Class (2)** | | | **Class (3)** |  |
|  | 0.0001 | 0.0331 | | | 0.9666 |  | 0.9999 | | 1.56e-07 | | | | 2.18e-13 |  | 0.9999 | 5.32e-05 | | 9.22e-09 |  | 0.0634 | | | 0.8166 | | | 0.1199 |  |
| **BPD** | 3.58 mm | | | | |  | 5.95 mm | | | | | | |  | 3.04 mm | | | |  | 3.04 mm | | | | | | |  |
| **MCD** | 1.45 mm | | | | |  | 4.58 mm | | | | | | |  | 2.4 mm | | | |  | 2.4 mm | | | | | | |  |
| **Tooth Site** | #47 | | | | | 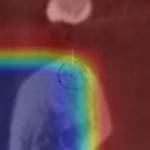 | #47 | | | | | | | 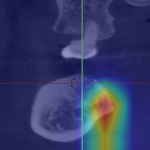 | #36 | | | | 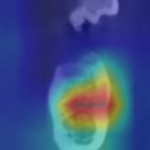 | #36 | | | | | | | 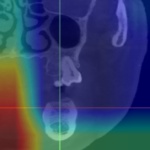 |
| **Implant Size** | 4.1*8 mm | | | | |  | 4.8*14 mm | | | | | | |  | 5.5*11.5 mm | | | |  | 5.5*11.5 mm | | | | | | |  |
| **Actual Risk** | Class (2): Caution | | | | |  | Class (2): Caution | | | | | | |  | Class (3): Risk | | | |  | Class (1): Safe | | | | | | |  |
| **Predicted Risk** | **BP** | | **MC** | | |  | **BP** | | | | **MC** | | |  | **BP** | | **MC** | |  | **BP** | | | | **MC** | | |  |
|  | - | | 2 | | |  | 2 | | | | 1 | | |  | - | | 3 | |  | 1 | | | | - | | |  |
| **Probabilities** | **Class (1)** | **Class (2)** | | | **Class (3)** |  | **Class (1)** | | **Class (2)** | | | | **Class (3)** |  | **Class (1)** | **Class (2)** | | **Class (3)** |  | **Class (1)** | | | **Class (2)** | | | **Class (3)** |  |
|  | 3.18e-10 | 0.9999 | | | 6.49e-08 |  | 3.28e-07 | | 0.9999 | | | | 2.88e-07 |  | 0.0031 | 0.0086 | | 0.9882 |  | 0.9999 | | | 1.75e-09 | | | 1.10e-14 |  |
| **BPD** | 5.21 mm | | | | |  | 2.22 mm | | | | | | |  | 4.08 mm | | | |  | 4.08 | | | | | | |  |
| **MCD** | 2.41 mm | | | | |  | 0 mm | | | | | | |  | 1.63 mm | | | |  | 1.63 | | | | | | |  |
| **Tooth Site** | #46 | | | | | 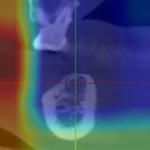 | #46 | | | | | | | 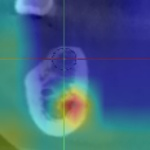 | #46 | | | | 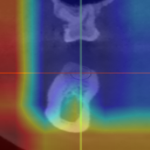 | #46 | | | | | | | 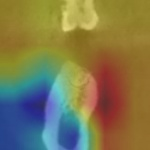 |
| **Implant Size** | 4.1*8 mm | | | | |  | 4.8*12 mm | | | | | | |  | 5.5*13 | | | |  | 4.8*14 mm | | | | | | |  |
| **Actual Risk** | Class (1): Safe | | | | |  | Class (3): Risk | | | | | | |  | Class (2): Caution | | | |  | Class (3): Risk | | | | | | |  |
| **Predicted Risk** | **BP** | | **MC** | | |  | **BP** | | | | **MC** | | |  | **BP** | | **MC** | |  | **BP** | | | | | **MC** | |  |
|  | - | | 1 | | |  | 3 | | | | - | | |  | 2 | | - | |  | 3 | | | | | - | |  |
| **Probabilities** | **Class (1)** | **Class (2)** | | | **Class (3)** |  | **Class (1)** | | **Class (2)** | | | | **Class (3)** |  | **Class (1)** | **Class (2)** | | **Class (3)** |  | **Class (1)** | | **Class (2)** | | | | **Class (3)** |  |
|  | 0.9999 | 2.78e-05 | | | 1.24e-09 |  | 9.02e-12 | | 1.38e-09 | | | | 0.9999 |  | 0.9762 | 0.0237 | | 2.29e-05 |  | 2.38E-05 | | 0.0005 | | | | 0.9994 |  |
| **BPD** | 3.25 mm | | | | |  | 0.5 mm | | | | | | |  | 2.61 mm | | | |  | 1.85 mm | | | | | | |  |
| **MCD** | 3.3 mm | | | | |  | 0 mm | | | | | | |  | 3.23mm | | | |  | 2.43 mm | | | | | | |  |
| **Tooth Site** | #46 | | | | | 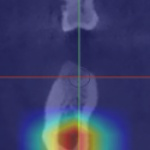 | #46 | | | | | | | 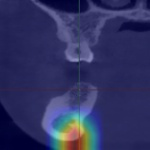 | #36 | | | | 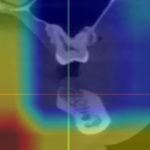 | #36 | | | | | | | 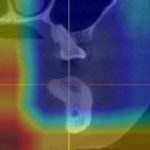 |
| **Implant Size** | 4.3*8.5 mm | | | | |  | 4.1*12 mm | | | | | | |  | 4.1*12 mm | | | |  | 4.3*11.5 mm | | | | | | |  |
| **Actual Risk** | Class (1): Safe | | | | |  | Class (3): Risk | | | | | | |  | Class (3): Risk | | | |  | Class (2): Caution | | | | | | |  |
| **Predicted Risk** | **BP** | | | **MC** | |  | **BP** | | | | **MC** | | |  | **BP** | | **MC** | |  | **BP** | | | | | **MC** | |  |
|  | - | | | 1 | |  | 3 | | | | - | | |  | 3 | | - | |  | 2 | | | | | - | |  |
| **Probabilities** | **Class (1)** | **Class (2)** | | | **Class (3)** |  | **Class (1)** | | **Class (2)** | | | | **Class (3)** |  | **Class (1)** | **Class (2)** | | **Class (3)** |  | **Class (1)** | | **Class (2)** | | | | **Class (3)** |  |
|  | 0.99999 | 3.31e-07 | | | 1.72e-12 |  | 6.11E-08 | | 1.89e-06 | | | | 0.9999 |  | 5.25e-07 | 1.03e-05 | | 0.9999 |  | 0.0123 | | 0.9805 | | | | 0.0071 |  |
| **BPD** | 3.3 mm | | | | |  | 1.71 mm | | | | | | |  | 0.25 mm | | | |  | 2.7 mm | | | | | | |  |
| **MCD** | 6.47 mm | | | | |  | 4.64 mm | | | | | | |  | 0.44 mm | | | |  | 4.06 mm | | | | | | |  |
| **Misprediction** | | | | | | | | | | | | | | | | | | | | | | | | | | | |
| **Tooth Site** | #46 | | | | | 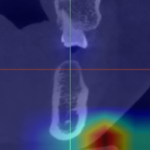 | #36 | | | | | | | 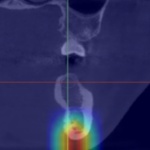 | #46 | | | | 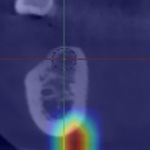 | #36 | | | | | | | 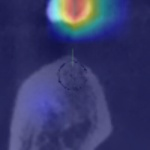 |
| **Implant Size** | 4.8*12 mm | | | | |  | 5*11.5 mm | | | | | | |  | 4.8*8 mm | | | |  | 4.1*12 mm | | | | | | |  |
| **Actual Risk** | Class (2): Caution | | | | |  | Class (2): Caution | | | | | | |  | Class (2): Caution | | | |  | Class (2): Caution | | | | | | |  |
| **Predicted Risk** | **BP** | | | **MC** | |  | **BP** | | | | **MC** | | |  | **BP** | | **MC** | |  | **BP** | | | | | **MC** | |  |
|  | 3 | | | - | |  | 1 | | | | - | | |  | 1 | | - | |  | - | | | | | 3 | |  |
| **Probabilities** | **Class (1)** | **Class (2)** | | | **Class (3)** |  | **Class (1)** | | **Class (2)** | | | | **Class (3)** |  | **Class (1)** | **Class (2)** | | **Class (3)** |  | **Class (1)** | | **Class (2)** | | | | **Class (3)** |  |
|  | 0.06342 | 0.1199 | | | 0.8166 |  | 0.8755 | | 0.1244 | | | | 2.99E-05 |  | 0.7769 | 0.2224 | | 0.0006 |  | 0.0003 | | 0 0.49028 | | | | 0.5904 |  |
| **BPD** | 2.11 mm | | | | |  | 2.71 mm | | | | | | |  | 2.8 mm | | | |  | 2.23 mm | | | | | | |  |
| **MCD** | 1.67 mm | | | | |  | 7.25 mm | | | | | | |  | 2.71 mm | | | |  | 2.68 mm | | | | | | |  |

A structured matrix of 24 representative implant sites was used to qualitatively evaluate DentaRisk-Net’s performance.

**Table S4.** Summary of studies on AI-driven methods for dental implant planning, risk assessment, and anatomical structure detection.

| **Study (short title)** | **First Author (Year)** | **AI Model / Architecture** | **Objective** | **Dataset / Sample Size** | **Key Results** | **Model Metrics (Accuracy / Dice / Sensitivity/ Specificity / time…etc.)** | **Contributions** |
| --- | --- | --- | --- | --- | --- | --- | --- |
| Automatic  presurgical implant planning | Elgarba et al. (2024)[5] | (Relu® Creator, Belgium) | Automated implant placement  compared to human intelligence (HI) | 10 CBCT + IOS | AI = 95% no major modification, HI = 96%; AI 2.2x faster | 95% placement accuracy, AI=198 ± 33s vs. HI= 435 ± 92s. AI consistency with zero-degree median surface deviation (MSD) compared to HI (MSD= 0.3 ± 0.17 mm) | Acceptable implant planning, and more time-efficient than HI |
| AI vs. HI in placement of dental implants | Elgarba et al. (2025)[6] | (Relu® Creator, Leuven, Belgium)[5]  3D CNN cloud-based platform | Validate automated AI tool for virtual implant placement vs expert planning | 50 matched CBCT + IOS scans; AI vs HI placement | AI= 89% vs 93% HI with 58% planning method unidentifiable in AI cases, AI > 2x faster | AI planning time 187s (34) vs. 406s (68) HI, AI MSD of zero vs intra-and inter-operator MSDs were 0.33 mm (0.14) and 0.56 mm (0.34), respectively. | Validated AI in selecting implant dimensions and positions with acceptable clinical outcomes |
| Deep learning for CBCT Planning | Bayrakdar et al. (2021)[7] | Deep CNN (Diagncat, San Francisco, USA) | Manual vs. AI planning, canal/sinus/missing tooth detection & measurements | 500+ CBCT | AI= 72.2% for canals detection, 66.4% for sinuses/fossae and 95.3% for missing tooth regions | 84.2% sucssessful bone height mesurments in (premolar, mandible) and (premolar and molar, maxilla) regions . manual vs AI (p > 0.05) | Detection of canal, sinus/fossa & missing teeth in CBCT with DL CNN |
| Enhanced tooth region detection (DL Models) | Al-Sarem et al. (2022)[8] | CNN models: AlexNet, VGG16/19, ResNet50, DenseNet169, MobileNetV3 | Missing teeth’s region detection on CBCT | 500 CBCT | Precision for DL models> 0.90  Best: DenseNet169 Precision=0.98, segmentation accuracy 93.3% w/out Seg Acc 89% | Precision of MobileNetV3= 0.95, VGG19= 0.94, ResNet50=0.94 , VGG16= 0.93, and AlexNet= 0.92 | Pretrained models benchmarking with and without segmentation |
| Deep learning bone segmentation | Al‑Asali et al. (2024)[9] | Two U-Net models | Segment missing tooth bone area & predict implant site | 150 CBCT | Dice = 93% Precision= 94%, Recall= 93% (implant site) | Dice 81%, Jaccard 68%, Precision 87%, Recall 75% (Segmentation), Dice 93%, Dice, Jaccard 88%, Precision 94%, Recall 93% (implant site) | High segmentation accuracy, clinically relevant predictions for implant area |
| Automatic placement of simulated dental in optimum positions | Alotaibi et al. (2025)[10] | YOLOv11 | Localize radiographic markers in CBCT for implants sites | 100 CBCT | For marker detection; F1-score = 0.59, MAE= 11.931-15.954, Classification accuracy for intra-osseous diameter= 0.76 , Intra-osseous length= 0.59 | F1-score = 0.59, , Precision = 0.59, Sensitivity= 0.60, mAP50= 0.56, mAP50-95= 0.33 | Focused on localization of fiducial markers |
| 2AI models for implant planning (Comparative study) | Roongruangsilpet al. (2025)[11] | Faster R-CNN  YOLOv7 | Compare 2 AI models performance using 4 implant planning software | 184 CBCT | Faster R-CNN performed better accuracy (Acc= 100) in PANO on DentiPlan Pro 3.7  YOLOv7 achieved higher detection rates in CBCT 93.75% on DentiPlan Pro 3.7 | CBCT (averaged across software):  - Faster R-CNN: Precision 57.5%, Recall 47.81%, F1-score 51.62%, JI 45.44%  - YOLOv7: Precision 39.06%, Recall 46.04%, F1-score 33.57%, JI 25.73%  PANO:  - Faster R-CNN: Precision 50%, Recall 43.96%, F1-score 42.65%, JI 34.9%  - YOLOv7: Precision 25.83%, Recall 28.13%, F1-score 22.14%, JI 16.46% | Compare Faster R-CNN and YOLOv7 across multiple CBCT imaging softwares for dental implant planning. |
| Automatic MC detection (DL CNN) | Kawk et al. (2020)[12] | 2D SegNet, 2D & 3D U-Nets | Mandibular canal segmentation via U-Net variants | 102 CBCT | 3D U-Net outperformed all models | 3D U-Net:  Global Accuracy = 0.999, Class Accuracy = 0.959, mIoU = 0.577  2D SegNet (pretrained): Global Accuracy = 0.962, Class Accuracy = 0.903, mIoU = 0.491  2D U-Net (adjacent slices):  Global Accuracy = 0.820, Class Accuracy = 0.683, mIoU = 0.411 2D U-Net (pretrained): Global Accuracy = 0.917, Class Accuracy = 0.591, mIoU = 0.460 | Comparison of 2D vs 3D networks in canal detection |
| Risk assessment of inferior alveolar nerve (IAN) injury | Picoli et al. (2023)[13] | (Relu BV, Leuven, Belgium) | Risk assessment of IAN injury via 3D AI-driven model with panoramic radiography (PANO) and (CBCT) | 25 Pt with PANO & CBCT scans (Within-patient Controlled Trail) | Sensitivity= 0.87 for 3D-AI and 0.89 for CBCT. | Sensitivity= 0.87 for 3D-AI, 0.89 for CBCT vs 0.73 for PANO  AUC=0.63 & Specificity=0.39 for 3D-AI, 0.58 and 0.28 for CBCT, and 0.57 and 0.41 for PANO | 3D-AI models demonstrated better performance metrics using CBCT over PANO |
| Assessment of likelihood of IAN injury | Gong et al. (2024)[14] | SS-TransUnet  CD-IAN | Enhance the precision of segmentation of MM3 and MC and classification accuracy IAN injury likelihood | 5374 OPG | SS-TransUnet:  MM3; Dice= 0.947, HD95= 12.969, Precision= 0.941  MC; Dice= 0.832, HD95= 17.706, Precision= 0.799.  CD-IAN injury class:  Precision= 0.843, Recall= 0.746, F1-score= 0.775, Accuracy= 0.846 | DL-based models:  Precision= 0.782 , Recall= 0.690, F1-score= 0.717, Accuracy= 0.808  (MM3 Detection)  Precision= 0.985, Recall= 0.989, F1-score= 0.987  (Segmentation)  MM3; Dice = 0.947, Precision= 0.941  MC; Dice = 0.832, Precision= 0.799 | Developed a three-step process for automatically assessing the likelihood of IAN injury |
| Analysis of DLS based localization of MC | Järnstedt et al. (2023)[15] | CNN U-net style architecture proposed by Jaskari et al [16] & Järnstedt et al [17] | DLS for automatic mandibular canal localization | 165 heterogeneous CBCT (72 Pt) | Likert RM Scoring; DLS: 0.877; Radiologist: 0.923 | Dice= 0.567 ±0.133  ASSD= 0.351 ± 0.135 mm  SMCD= 0.643 ± 0.186 mm  RC= 0.969 mm  Likert Score; DLS: 3.84 ± 0.65, Radiologist: 3.94 ± 0.27 | Evaluated temporal reproducibility of MC segmentation with DLS. |
| Comparison of 2D, 2.5D, and 3D segmentation for MC (CNNs) | Yang et al. (2025)[18] | 2D models:  2D-ResUNet, 2D-AttUNet  2.5D models: Axial–coronal–sagittal ResUNet and AttUNet  3D models:  3D-UNet, 3D-Swin UNETR | Mandibular canals segmentation from CBCT | 153 public CBCT  30 external CBCT | 3D-UNet outperformed all models on both datasets | 3D-Unet (Public):  Jaccard Index(JI)= 0.569 ± 0.107, Dice Similarity Coefficient (DSC)= 0.719 ± 0.092, Precision= 0.664 ± 0.131, Recall= 0.812 ± 0.095.  (External):  JI= 0.564 ± 0.092, DSC= 0.716 ± 0.081, Precision= 0.812 ± 0.087,  Recall= 0.652 ± 0.103 | Comprehensive comparison between 2D, 2.5D, and 3D CNN and transformer models on MC segmentation |
| Abnormal maxillary sinus diagnosis via object detection on DL | Zeng et al (2023)[19] | Detector:  YOLOv5  Diagnostor:  ResNet50 | Automated maxillary sinus abnormalities  detection | 2000 CBCT images (1000 Pt) (344 abnormalities) | Accuracy: >90%  Model outperformed Humans in diagnostic accuracy and speed | AUROC= 0.953, AUPRC= 0.882, Precision: 83.3%, Recall (Sensitivity): 87.0%, Specificity: 94.8%, F1-score= 0.85, Missed-diagnosis rate: 13% (as low as 2.17% with adjusted threshold), Misdiagnosis rate: 5.2% (as low as 3.25%)  Detector (YOLOv5):  Precision/Recall ≈ 100% for sinus localization.  Grad-CAM visualizations for model performance | AI-driven approach to support sinus evaluation in pre-implant planning that effectively isolates sinus regions and classifies abnormalities |
| Determination of a quantitative index sagittal root inclination | Lin et al. (2022)[20] | CNN model:  ResNeXt101 | (AI)-driven end-to-end CNN for measuring sagittal root inclination | 2,920 CBCT images (1,036 Pt) | Accuracy:  ±2.5° = 70%, ±5° = 93%, ±7.5° = 97.75%, ±10° = 98.75% | Prediction Error:  MAE = 2.16°, RMSE = 2.95°  Pearson coefficient= 0.915 (manual measurements)  Intraclass Correlation Coefficient (ICC):  Model vs human observers= 0.895–0.922  Prediction time = 0.001s per image (99.9% faster than manual measurement)  Heatmaps confirmed focus on correct anatomical regions | Automated end-to-end regression model for sagittal root inclination measurement without intermediate segmentation/landmark steps |

This table compiles findings from multiple studies that employed artificial intelligence in dental imaging and implantology. Collectively, these studies demonstrate advancements in automated implant placement, risk prediction, mandibular canal segmentation, maxillary sinus abnormality detection, and quantitative anatomical assessments, underscoring AI’s transformative potential in enhancing clinical workflows for dental implant planning.

**Table S5.** Comparison of TARs between AI predictions and human expert assessments for LPP and MCI.

| **BP** | | **TAR ≤ 0** | | **TAR ≤ 1** | | | | **TAR ≤ 2** | | | | |
| --- | --- | --- | --- | --- | --- | --- | --- | --- | --- | --- | --- | --- |
|  | | **AI** | **Human** | | **AI** | | **Human** | | **AI** | | | **Human** |
| Number of cases | | 30 | 30 | | 30 | | 30 | | 30 | | | 30 |
| Minimum | | 50.00 | 77.00 | | 54.00 | | 80.00 | | 80.00 | | | 100 |
| Maximum | | 100 | 100 | | 100 | | 100 | | 100 | | | 100 |
| Range | | 50.00 | 23.00 | | 46.00 | | 20.00 | | 20.00 | | | 0 |
| Mean | | 86.21 | 95.87 | | 92.23 | | 99.27 | | 95.67 | | | 100 |
| Std. Deviation | | 16.23 | 6.66 | | 13.89 | | 5.43 | | 4.03 | | | 0 |
| Std. Error of Mean | | 2.96 | 1.22 | | 2.54 | | 0.99 | | 0.74 | | | 0 |
| Lower 95% CI of mean | | 80.15 | 93.39 | | 88.81 | | 96.24 | | 97.5 | | | 100 |
| Upper 95% CI of mean | | 92.27 | 98.36 | | 99.19 | | 100.3 | | 100.5 | | | 100.7 |
| *p-*value | **TAR ≤ 0** |  |  | | < 0.001 | |  | | | 0.03 | | |
|  | **TAR ≤ 1** | < 0.001 |  | |  | |  | | | 0.001 | | |
|  | **TAR ≤ 2** | 0.03 |  | | 0.001 | |  | | |  | | |
| **MC** | | | | | | | | | | | | |
| Number of values | | 30 | 30 | | 30 | | 30 | | | 30 | | 30 |
| Minimum | | 40.00 | 60.00 | | 70.00 | | 90.00 | | | 80.00 | | 100 |
| Maximum | | 100 | 100 | | 100 | | 100 | | | 100 | | 100 |
| Range | | 60.00 | 40.00 | | 30.00 | | 10.00 | | | 10.00 | | 0 |
| Mean | | 86.72 | 94.30 | | 94.33 | | 99.63 | | | 96.33 | | 100 |
| Std. Deviation | | 15.24 | 9.95 | | 8.89 | | 1.83 | | | 2.54 | | 0 |
| Std. Error of Mean | | 2.78 | 1.82 | | 1.63 | | 0.33 | | | 0.46 | | 0 |
| Lower 95% CI of mean | | 81.03 | 90.59 | | 93.01 | | 98.95 | | | 98.39 | | 100 |
| Upper 95% CI of mean | | 92.42 | 98.02 | | 99.66 | | 100.3 | | | 100.4 | | 100.7 |
| *p-*value | **TAR ≤ 0** |  | | < 0.001 | | | | 0.001 | | | | |
|  | **TAR ≤ 1** | < 0.001 | |  | |  | | 0.02 | | | | |
|  | **TAR ≤ 2** | 0.001 | | 0.02 | | | |  | | |  | |

This table presents descriptive statistics for TAR analysis of AI predictions versus human expert assessments. Including: minimum, maximum, range, mean, standard deviation (SD), standard error (SE), and 95% confidence intervals (CI) for TAR thresholds ≤ 0 (perfect matches), ≤ 1 (off by no more than one class), and ≤ 2 (off by no more than two classes). *p*-values indicate significant differences ( *p* < 0.05) between AI and human assessments, with humans generally demonstrating higher TAR values across thresholds.

**References**

1. Rein, R.: Model Evaluation in Machine Learning Applications. In: Artificial Intelligence and Machine Learning in Sports Science. pp. 41–54. Springer Berlin Heidelberg, Berlin, Heidelberg (2025)

2. Varoquaux, G., Colliot, O.: Evaluating Machine Learning Models and Their Diagnostic Value. In: Neuromethods. pp. 601–630. Humana Press Inc. (2023)

3. Guilbert, T., Caelen, O., Chirita, A., Saerens, M.: Calibration methods in imbalanced binary classification. Ann Math Artif Intell. 92, 1319–1352 (2024). https://doi.org/10.1007/s10472-024-09952-8

4. Yin, S., Wang, L., Shafiq, M., Teng, L., Laghari, A.A., Khan, M.F.: G2Grad-CAMRL: An Object Detection and Interpretation Model Based on Gradient-Weighted Class Activation Mapping and Reinforcement Learning in Remote Sensing Images. IEEE J Sel Top Appl Earth Obs Remote Sens. 16, 3583–3598 (2023). https://doi.org/10.1109/JSTARS.2023.3241405

5. Elgarba, B.M., Fontenele, R.C., Mangano, F., Jacobs, R.: Novel AI-based automated virtual implant placement: Artificial versus human intelligence. J Dent. 147, (2024). https://doi.org/10.1016/j.jdent.2024.105146

6. Elgarba, B.M., Fontenele, R.C., Du, X., Mureșanu, S., Tarce, M., Meeus, J., Jacobs, R.: Artificial Intelligence Versus Human Intelligence in Presurgical Implant Planning: A Preclinical Validation. Clin Oral Implants Res. (2025). https://doi.org/10.1111/clr.14429

7. Bayrakdar, S.K., Orhan, K., Bayrakdar, I.S., Bilgir, E., Ezhov, M., Gusarev, M., Shumilov, E.: A deep learning approach for dental implant planning in cone-beam computed tomography images. BMC Med Imaging. 21, (2021). https://doi.org/10.1186/s12880-021-00618-z

8. Al-Sarem, M., Al-Asali, M., Alqutaibi, A.Y., Saeed, F.: Enhanced Tooth Region Detection Using Pretrained Deep Learning Models. Int J Environ Res Public Health. 19, (2022). https://doi.org/10.3390/ijerph192215414

9. Al-Asali, M., Alqutaibi, A.Y., Al-Sarem, M., Saeed, F.: Deep learning-based approach for 3D bone segmentation and prediction of missing tooth region for dental implant planning. Sci Rep. 14, (2024). https://doi.org/10.1038/s41598-024-64609-0

10. Alotaibi, S., Alsomali, M., Alghamdi, S., Alfadda, S., Alturaiki, I., Al-Ekrish, A., Altwaijry, N.: Automatic placement of simulated dental implants within CBCT images in optimum positions: a deep learning model. Med Biol Eng Comput. (2025). https://doi.org/10.1007/s11517-025-03327-9

11. Roongruangsilp, P., Narkbuakaew, W., Khongkhunthian, P.: Performance of two different artificial intelligence models in dental implant planning among four different implant planning software: a comparative study. BMC Oral Health. 25, (2025). https://doi.org/10.1186/s12903-025-06336-0

12. Kwak, G.H., Kwak, E.J., Song, J.M., Park, H.R., Jung, Y.H., Cho, B.H., Hui, P., Hwang, J.J.: Automatic mandibular canal detection using a deep convolutional neural network. Sci Rep. 10, (2020). https://doi.org/10.1038/s41598-020-62586-8

13. Picoli, F.F., Fontenele, R.C., Van der Cruyssen, F., Ahmadzai, I., Morgan, N., de-Azevedo-Vaz, S.L., Meeus, J., Willaert, R., Oliveira-Santos, N., Palla, B., Politis, C., Silva, M.A.G., Jacobs, R.: Risk assessment of inferior alveolar nerve injury after wisdom tooth removal using 3D AI-driven models: A within-patient study. J Dent. 139, (2023). https://doi.org/10.1016/j.jdent.2023.104765

14. Gong, Z., Feng, W., Su, X., Choi, C.: System for automatically assessing the likelihood of inferior alveolar nerve injury. Comput Biol Med. 169, (2024). https://doi.org/10.1016/j.compbiomed.2024.107923

15. Järnstedt, J., Sahlsten, J., Jaskari, J., Kaski, K., Mehtonen, H., Hietanen, A., Sundqvist, O., Varjonen, V., Mattila, V., Prapayasatok, S., Nalampang, S.: Reproducibility analysis of automated deep learning based localisation of mandibular canals on a temporal CBCT dataset. Sci Rep. 13, (2023). https://doi.org/10.1038/s41598-023-40516-8

16. Jaskari, J., Sahlsten, J., Järnstedt, J., Mehtonen, H., Karhu, K., Sundqvist, O., Hietanen, A., Varjonen, V., Mattila, V., Kaski, K.: Deep Learning Method for Mandibular Canal Segmentation in Dental Cone Beam Computed Tomography Volumes. Sci Rep. 10, (2020). https://doi.org/10.1038/s41598-020-62321-3

17. Järnstedt, J., Sahlsten, J., Jaskari, J., Kaski, K., Mehtonen, H., Lin, Z., Hietanen, A., Sundqvist, O., Varjonen, V., Mattila, V., Prapayasotok, S., Nalampang, S.: Comparison of deep learning segmentation and multigrader-annotated mandibular canals of multicenter CBCT scans. Sci Rep. 12, (2022). https://doi.org/10.1038/s41598-022-20605-w

18. Yang, S., Jeong, J.S., Song, D., Han, J.Y., Lim, S.-H., Kim, S., Yoo, J.-Y., Kim, J.-M., Kim, J.-E., Huh, K.-H., Lee, S.-S., Heo, M.-S., Yi, W.-J.: Comparison of 2D, 2.5D, and 3D segmentation networks for mandibular canals in CBCT images: a study on public and external datasets. BMC Oral Health. 25, 1126 (2025). https://doi.org/10.1186/s12903-025-06483-4

19. Zeng, P., Song, R., Lin, Y., Li, H., Chen, S., Shi, M., Cai, G., Gong, Z., Huang, K., Chen, Z.: Abnormal maxillary sinus diagnosing on CBCT images via object detection and ‘straight-forward’ classification deep learning strategy. J Oral Rehabil. 50, 1465–1480 (2023). https://doi.org/10.1111/joor.13585

20. Lin, Y., Shi, M., Xiang, D., Zeng, P., Gong, Z., Liu, H., Liu, Q., Chen, Z., Xia, J., Chen, Z.: Construction of an end-to-end regression neural network for the determination of a quantitative index sagittal root inclination. J Periodontol. 93, 1951–1960 (2022). https://doi.org/10.1002/JPER.21-0492
